# Child Developmental Patterns by Age 4 Years Across Subtypes of Hypertensive Disorders of Pregnancy

**DOI:** 10.1001/jamanetworkopen.2025.45719

**Published:** 2025-11-26

**Authors:** Geng Chen, Mami Ishikuro, Hisashi Ohseto, Aoi Noda, Genki Shinoda, Masatsugu Orui, Taku Obara, Shinichi Kuriyama

**Affiliations:** 1 Graduate School of Medicine, Tohoku University, Sendai, Japan; 2 Tohoku Medical Megabank Organization, Tohoku University, Sendai, Japan; 3 Tohoku University Hospital, Sendai, Japan; 4 International Research Institute of Disaster Science, Tohoku University, Sendai, Japan

## Abstract

**Importance:**

Exposure to hypertensive disorders of pregnancy (HDP) during the fetal stage has been linked to developmental delays in children. However, the associations between HDP subtypes and longitudinal patterns in child development remain unclear.

**Objective:**

To investigate the associations of HDP subtypes with child developmental patterns.

**Design, Setting, and Participants:**

This study used data from the Tohoku Medical Megabank Project Birth and Three-Generation Cohort Study, a prospective cohort study recruiting pregnant individuals in Japan between July 2013 and March 2017. Mother-child pairs were included in the analysis. The data analysis was conducted from November 2023 to February 2024.

**Exposures:**

HDP and its subtypes (gestational hypertension and preeclampsia) were identified using an algorithm applied to antenatal visit records.

**Main Outcomes and Measures:**

The latent class trajectory model was applied to child development scores at ages 6, 12, 24, 42, and 48 months to generate patterns in 5 domains (communication, gross motor, fine motor, problem solving, and personal-social). Multinomial Poisson regression analysis calculated the risk ratios (RRs) of different developmental patterns by exposure to any HDP as well as HDP subtypes. A subgroup analysis was performed by preterm birth.

**Results:**

Among 14 023 mother-child pairs (maternal mean [SD] age, 32.5 [4.8] years; 6754 [48.2%] female children), 1406 (10.0%) were exposed to HDP. Three patterns were identified in the 5 domains: normal, delay, and catch-up. Point estimates for delays in multiple domains of development were greater for any HDP, preeclampsia, and early-onset preeclampsia, but not all findings were statistically significant. Early-onset preeclampsia was associated with a significantly higher risk of delay pattern in problem solving domain (RR, 2.90; 95% CI, 1.43-5.89; adjusted *P* = .047), although the risks of delay pattern were not statistically significant in communication (RR, 1.94; 95% CI, 1.14-3.29; adjusted *P* = .15), gross motor (RR, 2.10; 95% CI, 1.26-3.51; adjusted *P* = .06), or fine motor (RR, 2.68; 95% CI, 1.26-5.71; adjusted *P* = .11) domains. In the term-born population, children exposed to preeclampsia and had an RR greater than 1 for the delay pattern in the problem solving domain (RR, 1.67; 95% CI, 1.92-2.74; adjusted *P* = .60), although this finding was not statistically significant after adjusting for multiple comparisons.

**Conclusions and Relevance:**

In this cohort study, fetal exposure to early-onset preeclampsia was associated with higher risk of a delayed child developmental pattern in the problem solving domain. The observations were modified by preterm birth. These findings suggest that children exposed to certain HDP subtypes during fetal life require developmental monitoring, especially if they were born preterm.

## Key Points

**Question:**

Are hypertensive disorders of pregnancy associated with child developmental patterns, and do associations vary by subtype?

**Findings:**

This cohort study among 14 023 mother-child pairs found that early-onset preeclampsia was associated with higher risks of delay in problem solving. This association was modified by preterm birth.

**Meaning:**

These findings suggest that among pregnant individuals with hypertensive disorders, expectant management should be adopted when possible and children exposed to preeclampsia in utero require monitoring in their progression in problem solving, irrespective of whether they were born at term.

## Teaser

This cohort study investigates the associations of fetal exposure to hypertensive disorders of pregnancy with child developmental patterns up to age 4 years.

## Introduction

Hypertensive disorders of pregnancy (HDP) are the most common complications of pregnancy, with a global prevalence of 116.4 per 100 000 women of childbearing age.^1^ HDP are associated with increased risks of mortality and morbidities in the offspring, and the risks vary among HDP subtypes. Among these subtypes, preeclampsia, one of the most severe complications of pregnancy, is associated with an increased risk of neurodevelopmental delay, as well as cardiovascular and metabolic diseases later in life.^2,3,4^

Child development is a dynamic process spanning from infancy to adulthood. It involves aspects of motor skills, speech and language, social, and performance and cognition.^5^ Some early developmental disorders can potentially persist into later life,^6^ while others dissipate over time.^7^ Although children may exhibit similar performance at certain stages of life, their underlying developmental trajectories may reflect distinct pathological mechanisms rooted in fetal exposures.

Prior studies that analyzed repeated measures of child development often conducted separate regression analyses at each time point, thereby overlooking continuity across time.^8^ Other studies applied mixed-effects models to examine associations with risk factors, yet these approaches lacked the ability to identify distinct developmental patterns.^9^ In contrast, latent class trajectory models (LCTMs) model no within-class variation, facilitating convergence and interpretability in the patterns.^10^ LCTMs have effectively identified patterns in human development and cognition,^11,12,13,14,15,16,17^ supporting their applicability to research on child development and behavior. Meanwhile, neurodevelopmental patterns during toddler and preschooler stages are strongly associated with language skills in their later life,^18^ highlighting the importance of focusing on early child development. Moreover, given the distinct pathological processes across HDP subtypes,^19,20,21^ it is plausible that these differences could induce varied patterns among exposed children in their development after birth.

This study aimed to determine the association of HDP and subtypes with developmental patterns in children. We hypothesized that patterns could be identified in child development and that the timing of HDP onset and HDP severity would determine the specific pattern.

## Methods

### Study Population

This study used data from the Tohoku Medical Megabank Project Birth and Three-Generation Cohort Study (TMM BirThree Cohort Study)—a prospective cohort study that recruited pregnant individuals from July 2013 to March 2017 in the Miyagi and Iwate Prefectures, Japan. The TMM BirThree Cohort Study continues to collect information on mother-child pairs and their families to contribute to precision medicine.^22,23,24^ The study protocol was approved by the Ethics Committee of the Tohoku Medical Megabank Organization on May 27, 2013. All participants provided written informed consent for inclusion in the study. This study followed the Strengthening the Reporting of Observational Studies in Epidemiology (STROBE) reporting guideline.

This study excluded mother-child pairs who met the following criteria: withdrawal of consent (625 participants), abortion (159 participants), stillbirths (185 participants), multiple pregnancies (624 participants), and missing HDP subtype (389 participants). To enable patterns in the developmental trajectory to be identified, we excluded participants with fewer than 2 responses to the Ages and Stages Questionnaires, third edition (ASQ-3) (7866 participants). We further excluded the participants with more than 30% of the variables missing (21 participants), which may lead to multiple imputation failure. Finally, 14 023 mother-child pairs were included in the analysis (eFigure 1 in Supplement 1).

### HDP Measures

Participant HDP subtypes were identified using an algorithm^25^ based on the diagnostic criteria of the American College of Obstetricians and Gynecologists, applied to their antennal visit records. Gestational hypertension (GH) was defined as systolic blood pressure 140 mm Hg or greater or diastolic blood pressure 90 mm Hg or greater, with no proteinuria, during at least 1 visit after gestational week 20 in individuals without history of high blood pressure. Chronic hypertension (CH) was defined as pregnant individuals who were hypertensive before 20 weeks of gestation or had a history of hypertensive disease before pregnancy. Preeclampsia was defined as GH with proteinuria (≥1 in the dipstick test) or other preeclampsia-related conditions after 20 gestational weeks. Superimposed preeclampsia was defined as hypertension complicated by proteinuria or other preeclampsia-related conditions before 20 weeks of gestation.

Within the preeclampsia subtype, participants were further classified based on the timing of proteinuria or other clinical symptoms: early-onset preeclampsia was defined as onset before 34 weeks of gestation; late-onset preeclampsia was defined as onset at or after 34 weeks of gestation. Subtypes of GH, preeclampsia, superimposed preeclampsia, and CH were categorized as HDP-exposed, while pregnant individuals without HDP were categorized as HDP-unexposed.

### Child Development Measures

The ASQ-3—a parent-reported developmental screening tool targeting 5 domains: communication, fine motor, gross motor, problem solving, and personal-social^26^—was used to assess children’s development. Each domain contains 6 questions about children’s daily activities, with responses of yes (10 points), sometimes (5 points), or not yet (0 points). Responses were summed into a domain score ranging from 0 to 60, with higher scores indicating better performance. The Japanese version of the ASQ-3, proven to be as effective as the original version,^27^ was completed by mothers when their children reached ages 6, 12, 24, 42, and 48 months.

### Covariates

Covariates were chosen based on previous studies,^8^ including maternal age at pregnancy (continuous in linear and quadratic effects), family annual income (<¥4 000 000, ¥4 000 000 to <¥6 000 000, ≥¥6 000 000; approximately <US $26 213, $26 213 to <$39 319, and ≥$39 319), maternal educational attainment (high school or lower, junior or vocational college, and university or higher), maternal prepregnancy body mass index (calculated as weight in kilograms divided by height in meters squared), parity (primipara, multipara), gestational diabetes (yes, no), maternal tobacco use in pregnancy (yes, no), maternal alcohol use in pregnancy (yes, no), maternal folic acid intake in pregnancy (no during pregnancy, yes during pregnancy, yes before and during pregnancy), and child sex (boy, girl). We imputed missing values in the covariates with multiple imputation with chained equations (eMethods and eTables 1-4 in Supplement 1).^28,29^

### Statistical Analysis

To identify the developmental patterns among the participant children, ASQ-3 scores at respective time points were *z*-score–scaled to eliminate artifactual effects caused by different questionnaire contents at each time point and fitted to LCTM.^30,31^ Linear, quadratic, or cubic effects of age at which the ASQ-3 were completed were applied to the fixed-effect model. Class numbers were given attempts, including 2, 3, 4, 5, 6, and 7. We further assessed the model adequacies: the average maximum posterior probability of assignments (APPA) was calculated to ensure a balanced classification, and the odds of correct classification (OCC) were calculated to ensure the overall classification accuracy. The thresholds for good modeling were set as APPA greater than 70% and OCC greater than 4.0. Subsequently, the optimal model was selected based on the lowest Bayesian information criterion (BIC).

Baseline characteristics and developmental patterns were compared between the HDP-exposed and HDP-unexposed groups using χ^2^ tests and analysis of variance. We contrasted the rates of different developmental patterns by multinomial Poisson regression analysis, adjusted for covariates, in the following groups: (1) HDP vs non-HDP, (2) GH vs non-HDP, (3) preeclampsia vs non-HDP, (4) early-onset preeclampsia vs non-HDP, and (5) late-onset preeclampsia vs non-HDP. Risk ratios (RRs) were calculated along with 95% CIs. Analyses were conducted independently in 10 imputed datasets, then pooled under the Rubin rule. Due to the reported significant association between preterm births and neurodevelopmental disorders,^32^ we conducted a subgroup analysis by preterm birth. Collected from the medical records at delivery, gestational age younger than 37 weeks was considered preterm birth.

We performed 3 sensitivity analyses. First, we excluded participants with missing covariates and repeated the analysis in a complete-case dataset (n = 11 774). Second, we excluded children with congenital abnormalities at birth or age 1 month (eTable 6 in Supplement 1). Diagnoses were obtained from the medical records from health checkups. Third, to compare with previous literature results, we regressed the ASQ-3 *z*-scores to time points and each HDP subtype using linear mixed-effect models.

All analyses were performed using R software version 4.1.2 (R Project for Statistical Computing).^33^ The data analysis was conducted from November 2023 to February 2024. In the regression analysis, *P* values were adjusted for multiple comparisons within each exposure, using the Hochberg procedure.^34^ P values were 2-sided, and *P* < .05 was considered statistically significant.

## Results

### Participant Characteristics

Among 14 023 mother-child pairs (maternal mean [SD] age, 32.5 [4.8] years; 6754 [48.2%] female children), 1406 (10.0%) were exposed to HDP, of whom 552 (39.3%) were exposed to GH, 328 (23.3%) exposed to CH, 349 (24.8%) exposed to preeclampsia, and 177 (12.6%) exposed to superimposed preeclampsia. Within the preeclampsia subtype, 84 pregnant individuals had early-onset preeclampsia, and 265 pregnant individuals had late-onset preeclampsia. Participants with HDP were more likely to have BMI greater than 25 and be older, primiparas, and educated below high school level, with low family annual income and with gestational diabetes during pregnancy. Their children were more likely to be born preterm (Table 1). Cohort characteristics are presented among the HDP subtypes in eTable 1 in Supplement 1.

**Table 1.** Baseline Characteristics of Mother-Child Pairs According to HDP Prevalence

Characteristics	Mother-child pairs, No. (%)			*P* value
	Total (N = 14 023)	HDP		
		Unexposed (n = 12 617)	Exposed (n = 1406)	
Maternal age, mean (SD), y	32.5 (4.8)	32.5 (4.8)	33.1 (5.2)	<.001
Family income, ¥/y^a^				
<4 000 000	4903 (35.0)	4356 (34.5)	547 (38.9)	.003
≥4 000 000 to <6 000 000	4647 (33.1)	4193 (33.2)	454 (32.3)	
≥6 000 000	4473 (31.9)	4068 (32.2)	405 (28.8)	
Maternal educational level				
≤High school	4576 (32.6)	4015 (31.8)	561 (39.9)	<.001
Junior or vocational college	5424 (38.7)	4891 (38.8)	533 (37.9)	
≥University	4023 (28.7)	3711 (29.4)	312 (22.2)	
Maternal prepregnancy BMI				
<18.5	10 451 (74.5)	9593 (76.0)	858 (61.0)	<.001
≥18.5 to <25	1841 (13.1)	1712 (13.6)	129 (9.2)	
≥25	1731 (12.3)	1312 (10.4)	419 (29.8)	
Multiparous	7405 (52.8)	6778 (53.7)	627 (44.6)	<.001
Gestational diabetes	328 (2.3)	275 (2.2)	53 (3.8)	<.001
Maternal tobacco use	1898 (13.5)	1695 (13.4)	203 (14.4)	.32
Maternal alcohol use	3064 (21.8)	2767 (21.9)	297 (21.1)	.51
Maternal folic acid intake				
No during pregnancy	5290 (37.7)	4767 (37.8)	523 (37.2)	.47
Yes during pregnancy	6217 (44.3)	5603 (44.4)	614 (43.7)	
Yes before and during pregnancy	2516 (17.9)	2247 (17.8)	269 (19.1)	
Preterm birth	715 (5.1)	580 (4.6)	135 (9.6)	<.001
Child sex				
Female	6754 (48.2)	6085 (48.2)	669 (47.6)	.67
Male	7269 (51.8)	6532 (51.8)	737 (52.4)	
HDP subtypes				
Gestational hypertension	552 (3.9)	NA	552 (39.3)	NA
Chronic hypertension	328 (2.3)	NA	328 (23.3)	
Preeclampsia				
Any	349 (2.5)	NA	349 (24.8)	
Early onset	84 (0.6)	NA	84 (6.0)	
Late onset	265 (1.9)	NA	265 (18.8)	
Superimposed preeclampsia	177 (1.3)	NA	177 (12.6)	

Abbreviations: HDP, hypertensive disorders of pregnancy; BMI, body mass index (calculated as weight in kilograms divided by height in meters squared); NA, not applicable.

^a^ Approximately <US $26 213, $26 213 to <$39 319, and ≥$39 319, respectively.

### Child Developmental Patterns

After model adequacies were assessed by APPA and OCC (eTable 5 in Supplement 1), the linear model classifying the participant children into 3 classes by LCTM was optimal, with the lowest BICs in 4 domains (communication, 145 346.2451; gross motor, 143 839.1751; fine motor, 147 718.4676; problem solving, 142 704.6957; personal-social, 146 730.775). In the fine motor domain, since no model met the OCC threshold, we adopted the same model identified as optimal in the other domains. This model yielded the lowest BIC (147 718.4616), APPAs above the threshold, and acceptable OCC values. The Figure shows the estimation graphs of the 3 developmental patterns in the domains of communication, gross motor, fine motor, problem solving, and personal-social. According to the variation in *z*-score trends with time progression in each pattern, we name the 3 patterns normal, catch-up, and delay. For each pattern in the 5 domains, the actual mean and SD values of ASQ-3 scores were in accordance with the estimation graphs (eFigure 2 in Supplement 1). Children in the catch-up pattern initially showed delay signs but reached behavioral milestones at very close to the expected rates by age 48 months. Children in the delay pattern had persistent delay performances from toddler to early childhood.

**Figure.**
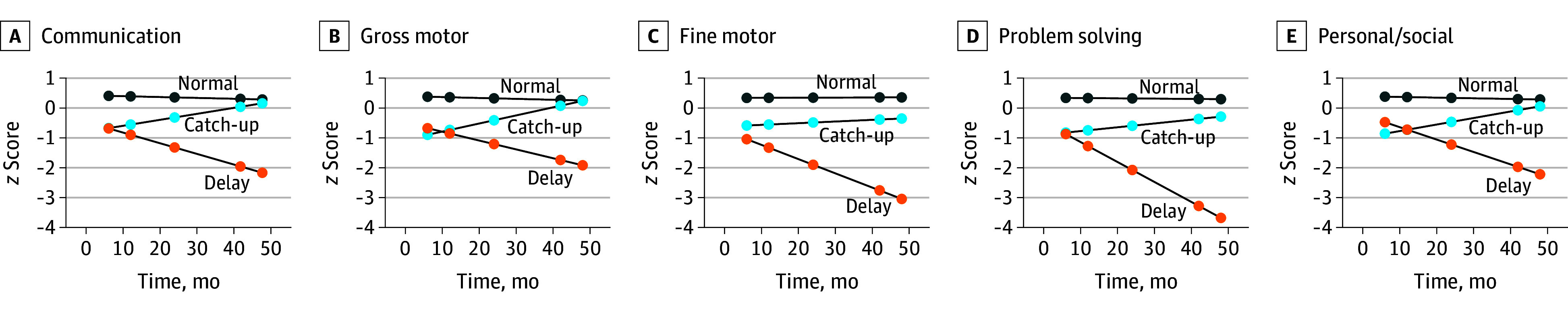
Estimated Trajectory of Developmental Patterns in 5 Domains Identified Using a Latent Class Trajectory Model Three patterns were attributed to the participants based on their growing trends in the Ages and Stages Questionnaires, third edition, *z*-score: normal (stayed above the mean from ages 6 to 48 months), catch-up (distant from no delay at age 6 months, almost at the normal level at age 48 months), and delay (distant from normal from ages 6 to 48 months).

The normal pattern described the most children, with 66.1% to 74.9% of children reaching behavior milestones at the expected rate, followed by catch-up (18.9%-26.8%) and delay (3.0%-9.1%). These proportions significantly differed between HDP-exposed and HDP-unexposed participants. Children unexposed to HDP in utero were more likely to be in the normal pattern across all 5 domains (Table 2).

**Table 2.** Developmental Pattern Distribution Across HDP and Subtypes

Domain and pattern	Mother-child pairs, No. (%)			*P* value	Mother-child pairs, No. (%) by HDP subtype				
	Total (N = 14 023)	HDP							
		Unexposed (n = 12 617)	Exposed (n = 1406)		CH (n = 328)	GH (n = 552)	PE (n = 349)	PE subtypes	
								EO (n = 84)	LO (n = 265)
Communication									
Normal	9273 (66.1)	8380 (66.4)	893 (63.5)	.007	211 (64.3)	361 (65.4)	213 (61.0)	42 (50.0)	171 (64.5)
Catch-up	3517 (25.1)	3158 (25.0)	359 (25.5)		76 (23.2)	138 (25.0)	94 (26.9)	28 (33.3)	66 (24.9)
Delay	1233 (8.8)	1079 (8.6)	154 (11.0)		41 (12.5)	53 (9.6)	42 (12.0)	14 (16.7)	28 (10.6)
Gross motor									
Normal	10 096 (72.0)	9133 (72.4)	963 (68.5)	<.001	227 (69.2)	388 (70.3)	227 (65.0)	46 (54.8)	181 (68.3)
Catch-up	2650 (18.9)	2377 (18.8)	273 (19.4)		64 (19.5)	102 (18.5)	74 (21.2)	23 (27.4)	51 (19.2)
Delay	1276 (9.1)	1106 (8.8)	170 (12.1)		37 (11.3)	62 (11.2)	48 (13.8)	15 (17.9)	33 (12.5)
Fine motor									
Normal	9835 (70.1)	8911 (70.6)	924 (65.7)	.001	215 (65.5)	361 (65.4)	226 (64.8)	49 (58.3)	177 (66.8)
Catch-up	3763 (26.8)	3334 (26.4)	429 (30.5)		97 (29.6)	177 (32.1)	105 (30.1)	28 (33.3)	77 (29.1)
Delay	421 (3.0)	368 (2.9)	53 (3.8)		16 (4.9)	14 (2.5)	18 (5.2)	7 (8.3)	11 (4.2)
Problem solving									
Normal	10 507 (74.9)	9519 (75.4)	988 (70.3)	<.001	224 (68.3)	398 (72.1)	237 (68.1)	46 (54.8)	191 (72.3)
Catch-up	3051 (21.8)	2691 (21.3)	360 (25.6)		89 (27.1)	136 (24.6)	89 (25.6)	30 (35.7)	59 (22.3)
Delay	450 (3.2)	393 (3.1)	57 (4.1)		15 (4.6)	18 (3.3)	22 (6.3)	8 (9.5)	14 (5.3)
Personal-social									
Normal	9838 (70.2)	8897 (70.5)	941 (66.9)	.011	215 (65.5)	375 (67.9)	227 (65.0)	46 (54.8)	181 (68.3)
Catch-up	3235 (23.1)	2886 (22.9)	349 (24.8)		82 (25.0)	136 (24.6)	90 (25.8)	30 (35.7)	60 (22.6)
Delay	940 (6.7)	824 (6.5)	116 (8.3)		31 (9.5)	41 (7.4)	32 (9.2)	8 (9.5)	24 (9.1)

Abbreviations: HDP, hypertensive disorders of pregnancy; CH, chronic hypertension; GH, gestational hypertension; PE, preeclampsia; EO, early onset; LO, late onset.

### HDP and Risk of Developmental Patterns

After adjusting for covariates, compared with HDP-unexposed children, HDP-exposed children had RRs greater than 1 for the catch-up pattern in fine motor (RR, 1.10; 95% CI, 1.00-1.22; adjusted *P* = .78) and problem solving (RR, 1.12; 95% CI, 1.00-1.26; adjusted *P* = .65) domains and for the delay pattern in the gross motor domain (RR, 1.25; 95% CI, 1.06-1.48; adjusted *P* = .11), although these findings were not statistically significant after adjusting for multiple comparisons (Table 3). In the term-birth or preterm birth populations, HDP-exposed children had no higher risk of any adverse pattern in any of the 5 domains (Table 4). In the term-birth population, HDP-exposed children’s RRs for catch-up and delay patterns were lower than in the full population (Table 4). However, after *P* value corrections, the risks observed were not statistically significant (Table 3 and Table 4).

**Table 3.** aRRs of Child Developmental Patterns in HDP Overall and Its Subtypes

Domain	HDP overall (n = 1406)		GH (n = 552)		PE (n = 349)		EO PE (n = 84)		LO PE (n = 265)	
	aRR (95% CI)^a^	*P* value^b^	aRR (95% CI)^a^	*P* value^b^	aRR (95% CI)^a^	*P* value^b^	aRR (95% CI)^a^	*P* value^b^	aRR (95% CI)^a^	*P* value^b^
**Communication**										
Catch-up vs normal	1.05 (0.94-1.18)	.91	1.02 (0.86-1.21)	.97	1.15 (0.93-1.41)	.78	1.51 (1.04-2.19)	.25	1.04 (0.82-1.33)	.91
Delay vs normal	1.15 (0.97-1.37)	.91	1.04 (0.78-1.37)	.97	1.35 (0.99-1.83)	.73	1.94 (1.14-3.29)	.15	1.17 (0.80-1.70)	.91
Catch-up vs delay	0.97 (0.86-1.08)	.91	0.99 (0.84-1.18)	.97	0.94 (0.76-1.16)	.78	0.93 (0.64-1.35)	.85	0.95 (0.74-1.21)	.91
**Gross motor**										
Catch-up vs normal	1.05 (0.92-1.19)	.91	1.00 (0.82-1.22)	.97	1.17 (0.93-1.47)	.78	1.62 (1.07-2.44)	.20	1.04 (0.79-1.37)	.91
Delay vs normal	1.25 (1.06-1.48)	.11	1.19 (0.92-1.54)	.97	1.51 (1.13-2.02)	.08	2.10 (1.26-3.51)	.06	1.34 (0.95-1.90)	.91
Catch-up vs delay	0.93 (0.82-1.06)	.91	0.93 (0.77-1.14)	.97	0.91 (0.72-1.15)	.78	0.91 (0.60-1.38)	.85	0.91 (0.69-1.21)	.91
**Fine motor**										
Catch-up vs normal	1.10 (1.00-1.22)	.78	1.15 (0.99-1.34)	.97	1.12 (0.92-1.36)	.78	1.26 (0.87-1.83)	.85	1.08 (0.86-1.35)	.91
Delay vs normal	1.10 (0.82-1.49)	.91	0.80 (0.47-1.36)	.97	1.66 (1.03-2.67)	.50	2.68 (1.26-5.71)	.11	1.34 (0.73-2.44)	.91
Catch-up vs delay	1.01 (0.91-1.12)	.91	1.04 (0.89-1.21)	.97	0.96 (0.79-1.16)	.78	0.90 (0.62-1.31)	.85	0.98 (0.78-1.23)	.91
**Problem solving**										
Catch-up vs normal	1.12 (1.00-1.26)	.65	1.09 (0.92-1.29)	.97	1.17 (0.95-1.45)	.78	1.66 (1.16-2.38)	.08	1.02 (0.78-1.32)	.91
Delay vs normal	1.08 (0.81-1.44)	.91	0.90 (0.56-1.46)	.97	1.79 (1.16-2.76)	.12	2.90 (1.43-5.89)	.047	1.48 (0.86-2.53)	.91
Catch-up vs delay	1.01 (0.90-1.13)	.91	1.02 (0.86-1.22)	.97	0.93 (0.75-1.15)	.78	0.92 (0.64-1.32)	.85	0.94 (0.72-1.21)	.91
**Personal-social**										
Catch-up vs normal	1.08 (0.97-1.21)	.91	1.08 (0.91-1.28)	.97	1.17 (0.95-1.44)	.78	1.62 (1.13-2.33)	.10	1.03 (0.80-1.33)	.91
Delay vs normal	1.09 (0.89-1.33)	.91	0.99 (0.72-1.36)	.97	1.32 (0.92-1.88)	.78	1.53 (0.76-3.08)	.85	1.26 (0.84-1.90)	.91
Catch-up vs delay	0.99 (0.88-1.11)	.91	1.00 (0.84-1.19)	.97	0.97 (0.78-1.20)	.78	1.04 (0.72-1.49)	.85	0.94 (0.73-1.22)	.91

Abbreviations: aRR, adjusted risk ratio; EO, early onset; GH, gestational hypertension; HDP, hypertensive disorders of pregnancy; LO, late onset; PE, preeclampsia.

^a^ Reference group: HDP-unexposed. Adjusted for maternal age at pregnancy (linear and quadratic effects), family annual income, maternal educational attainment, maternal prepregnancy body mass index, parity, gestational diabetes prevalence, maternal tobacco use during pregnancy, maternal alcohol use during pregnancy, maternal folic acid intake during pregnancy, and the child’s sex.

^b^ Adjusted for multiple comparisons using the Hochberg procedure.

**Table 4.** Relative Risks of Child Developmental Patterns in HDP Overall and Its Subtypes in the Term-Birth and Preterm Birth Populations

Domain	HDP		GH		PE		EO PE		LO PE	
	aRR (95% CI)^a^	*P* value^b^	aRR (95% CI)^a^	*P* value^b^	aRR (95% CI)^a^	*P* value^b^	aRR (95% CI)^a^	*P* value^b^	aRR (95% CI)^a^	*P* value^b^
**Term birth**										
No.	1270	NA	513	NA	302	NA	56	NA	246	NA
Communication										
Catch-up vs normal	1.02 (0.90-1.15)	.97	1.01 (0.85-1.21)	.99	1.08 (0.86-1.35)	.92	1.46 (0.92-2.32)	.97	1.00 (0.77-1.29)	.99
Delay vs normal	1.08 (0.89-1.30)	.97	0.96 (0.71-1.31)	.99	1.20 (0.84-1.71)	.92	1.71 (0.85-3.44)	.97	1.09 (0.73-1.64)	.99
Catch-up vs delay	0.97 (0.86-1.10)	.97	1.01 (0.85-1.21)	.99	0.95 (0.76-1.20)	.92	0.96 (0.60-1.53)	.97	0.96 (0.74-1.24)	.99
Gross motor										
Catch-up vs normal	0.99 (0.86-1.14)	.97	0.95 (0.77-1.18)	.99	1.05 (0.81-1.37)	.92	1.15 (0.65-2.03)	.97	1.03 (0.77-1.38)	.99
Delay vs normal	1.14 (0.95-1.37)	.97	1.12 (0.84-1.48)	.99	1.17 (0.82-1.68)	.92	0.85 (0.32-2.26)	.97	1.25 (0.85-1.83)	.99
Catch-up vs delay	0.95 (0.83-1.09)	.97	0.94 (0.76-1.16)	.99	0.98 (0.75-1.28)	.92	1.15 (0.65-2.03)	.97	0.95 (0.71-1.27)	.99
Fine motor										
Catch-up vs normal	1.09 (0.98-1.22)	.97	1.15 (0.98-1.35)	.99	1.09 (0.88-1.35)	.92	1.07 (0.66-1.76)	.97	1.10 (0.87-1.39)	.99
Delay vs normal	0.99 (0.71-1.40)	.97	0.83 (0.46-1.47)	.99	1.27 (0.69-2.32)	.92	1.18 (0.29-4.75)	.97	1.30 (0.67-2.52)	.99
Catch-up vs delay	1.01 (0.91-1.13)	.97	1.04 (0.88-1.22)	.99	0.99 (0.80-1.23)	.92	0.99 (0.60-1.62)	.97	0.99 (0.78-1.25)	.99
Problem solving										
Catch-up vs normal	1.05 (0.93-1.19)	.97	1.04 (0.87-1.26)	.99	1.03 (0.80-1.32)	.92	1.16 (0.68-1.96)	.97	1.00 (0.76-1.32)	.99
Delay vs normal	1.02 (0.74-1.40)	.97	0.86 (0.51-1.45)	.99	1.67 (1.02-2.74)	.60	2.22 (0.83-6.00)	.97	1.56 (0.89-2.73)	.99
Catch-up vs delay	1.00 (0.89-1.14)	.97	1.02 (0.85-1.23)	.99	0.92 (0.72-1.18)	.92	0.90 (0.53-1.53)	.97	0.92 (0.70-1.22)	.99
Personal-social										
Catch-up vs normal	1.03 (0.91-1.17)	.97	1.04 (0.87-1.26)	.99	1.01 (0.79-1.30)	.92	1.07 (0.62-1.85)	.97	1.00 (0.76-1.32)	.99
Delay vs normal	0.99 (0.80-1.24)	.97	0.93 (0.66-1.30)	.99	1.18 (0.79-1.76)	.92	0.77 (0.25-2.40)	.97	1.27 (0.83-1.94)	.99
Catch-up vs delay	1.00 (0.88-1.13)	.97	1.00 (0.83-1.20)	.99	0.96 (0.75-1.23)	.92	1.08 (0.62-1.87)	.97	0.93 (0.71-1.23)	.99
**Preterm birth**										
No.	135	NA	39	NA	39	NA	28	NA	19	NA
Communication										
Catch-up vs normal	1.16 (0.83-1.60)	>.99	1.01 (0.55-1.83)	.98	1.28 (0.78-2.09)	.88	1.20 (0.63-2.29)	.88	1.44 (0.69-2.98)	>.99
Delay vs normal	1.40 (0.88-2.22)	>.99	1.74 (0.84-3.62)	.98	1.55 (0.79-3.05)	.88	1.48 (0.64-3.45)	.88	1.71 (0.59-4.92)	>.99
Catch-up vs delay	0.95 (0.68-1.33)	>.99	0.85 (0.46-1.54)	.98	0.91 (0.55-1.52)	.88	0.92 (0.47-1.80)	.88	0.92 (0.44-1.91)	>.99
Gross motor										
Catch-up vs normal	1.19 (0.85-1.67)	>.99	1.26 (0.72-2.23)	.98	1.46 (0.87-2.46)	.88	1.93 (1.04-3.58)	.55	0.95 (0.38-2.36)	>.99
Delay vs normal	1.48 (0.98-2.22)	>.94	1.60 (0.80-3.17)	.98	2.01 (1.16-3.47)	.19	2.47 (1.27-4.82)	.12	1.54 (0.64-3.72)	>.99
Catch-up vs delay	0.86 (0.60-1.22)	>.99	0.89 (0.50-1.57)	.98	0.78 (0.46-1.31)	.88	0.79 (0.42-1.47)	.88	0.74 (0.30-1.82)	>.99
Fine motor										
Catch-up vs normal	0.98 (0.72-1.33)	>.99	1.02 (0.63-1.68)	.98	0.94 (0.57-1.53)	.88	1.11 (0.62-2.00)	.88	0.71 (0.31-1.63)	>.99
Delay vs normal	0.95 (0.50-1.82)	>.99	0.42 (0.10-1.83)	.98	1.59 (0.69-3.63)	.88	1.83 (0.68-4.91)	.88	1.17 (0.26-5.15)	>.99
Catch-up vs delay	1.00 (0.73-1.35)	>.99	1.11 (0.68-1.81)	.98	0.90 (0.55-1.48)	.88	0.90 (0.49-1.63)	.88	0.92 (0.40-2.12)	>.99
Problem solving										
Catch-up vs normal	1.31 (0.97-1.76)	>.99	1.31 (0.80-2.12)	.98	1.42 (0.91-2.20)	.88	1.68 (1.00-2.82)	.66	1.06 (0.49-2.30)	>.99
Delay vs normal	1.00 (0.49-2.05)	>.99	1.12 (0.33-3.84)	.98	1.41 (0.53-3.73)	.88	1.77 (0.58-5.42)	.88	0.82 (0.11-6.30)	>.99
Catch-up vs delay	1.10 (0.81-1.49)	>.99	1.09 (0.67-1.77)	.98	1.07 (0.68-1.69)	.88	1.04 (0.61-1.78)	.88	1.13 (0.50-2.53)	>.99
Personal-social										
Catch-up vs normal	1.08 (0.80-1.45)	>.99	1.14 (0.70-1.85)	.98	1.29 (0.85-1.98)	.88	1.54 (0.93-2.55)	.88	0.97 (0.47-2.01)	>.99
Delay vs normal	1.52 (0.90-2.56)	>.99	1.63 (0.66-4.01)	.98	1.84 (0.81-4.15)	.88	2.54 (0.97-6.66)	.72	1.28 (0.29-5.64)	>.99
Catch-up vs delay	0.93 (0.69-1.26)	>.99	0.98 (0.60-1.59)	.98	0.97 (0.63-1.49)	.88	0.95 (0.57-1.58)	.88	1.00 (0.48-2.08)	>.99

Abbreviations: HDP, hypertensive disorders of pregnancy; PE, preeclampsia; GH, gestational hypertension; EO, early onset; LO, late onset; RR, risk ratio.

^a^ Reference group: HDP-unexposed. Adjusted for maternal age at pregnancy (linear and quadratic effects), family annual income, maternal educational attainment, maternal prepregnancy body mass index, parity, gestational diabetes prevalence, maternal tobacco use during pregnancy, maternal alcohol use during pregnancy, maternal folic acid intake during pregnancy, and the child’s sex.

^b^ Adjusted for multiple comparisons using the Hochberg procedure.

### GH and Risk of Developmental Patterns

GH was not associated with elevated risk of adverse developmental pattern in the overall population (Table 3) or in the term-birth or preterm birth populations (Table 4). In the term-birth population, GH-exposed children’s RRs for the catch-up and delay patterns were lower than in the full population, except for the fine motor domain (Table 4).

### Preeclampsia and Risk of Developmental Patterns

Preeclampsia-exposed children had higher RRs for the delay pattern in the gross motor (RR, 1.51; 95% CI, 1.13-2.02; adjusted *P* = .08), fine motor (RR, 1.66; 95% CI, 1.03-2.67; adjusted *P* = .50), and problem solving (RR, 1.79; 95% CI, 1.16-2.76; adjusted *P* = .12) domains (Table 3). In the term-birth population, preeclampsia-exposed children’s RRs for the catch-up and delay patterns were lower than in the full population, but the RR was higher for a delay pattern in the problem solving domain (RR, 1.67; 95% CI, 1.02-2.74; adjusted *P* = .60) (Table 4). In the preterm birth population, preeclampsia-exposed children had an RR of 2.01 (95% CI, 1.16-3.47) for the delay pattern in gross motor domain (Table 4). After adjusting *P* values using the Hochberg procedure, only the association between early-onset preeclampsia and delay pattern in the problem solving domain remained statistically significant (RR, 2.90; 95% CI, 1.43-5.89; adjusted *P* = .047).

In the preeclampsia subtypes according to onset, early-onset preeclampsia–exposed children had higher RRs for the catch-up pattern in communication (RR, 1.51; 95% CI, 1.04-2.19; adjusted *P* = .25), gross motor (RR, 1.62; 95% CI, 1.07-2.44; adjusted *P* = .20), problem solving (RR, 1.66; 95% CI, 1.16-2.38; adjusted *P* = .08), and personal-social (RR, 1.62; 95% CI, 1.13-2.33; adjusted *P* = .10) domains. Their RRs for the delay pattern in communication (RR, 1.94; 95% CI, 1.14-3.29; adjusted *P* = .15), gross motor (RR, 2.10; 95% CI, 1.26-3.51; adjusted *P* = .06), fine motor (RR, 2.68; 95% CI, 1.26-5.71; adjusted *P* = .11), and problem solving (RR 2.90, 95% CI 1.43-5.89; adjusted *P* = .047) domains were approximately 2- to 3-fold higher relative to a normal pattern. If early-onset preeclampsia–exposed children were born at term, they had no significant risk of any adverse pattern in any domain (Table 4). In the preterm population, RRs greater than 1 were observed in fewer domains with higher estimates: catch-up pattern in gross motor (RR, 1.93; 95% CI, 1.04-3.58; adjusted *P* = .55) and problem solving (RR, 1.68; 95% CI, 1.00-2.82; adjusted *P* = .66) domains and delay pattern in the gross motor domain (RR, 2.47; 95% CI, 1.27-4.82; adjusted *P* = .12) (Table 4), although they were not statistically significant after adjustment. No associations in developmental patterns were observed among the late-onset preeclampsia–exposed children (Table 3 and Table 4)

### Sensitivity Analysis

In the complete-case population (eTable 8 in Supplement 1), the RRs were of generally consistent directions as the main analysis (Table 3), with 2 exceptions. In the congenital abnormality-free population, the RRs were generally consistent (eTable 8 in Supplement 1).

In the linear mixed-effect models, across 5 domains, children exposed to HDP and subtypes showed significantly lower *z*-scores at the baseline. The trend of *z*-scores over time was not materially different within the exposed and unexposed groups (eTable 8 in Supplement 1).

## Discussion

In this study, we identified 3 child developmental patterns: normal, delay, and catch-up, and clarified the roles of exposure to any HDP and to its subtypes in developmental patterns in children. Children with exposure to the early-onset preeclampsia subtype had a 2- to 3-fold higher RRs for the delay pattern in multiple domains. In term-born children, the association elevated RRs were only observed for preeclampsia and a delay pattern in the problem solving domain. In the preterm population, children exposed to preeclampsia and early-onset preeclampsia had higher RRs for development catch-up and delay.

Previous studies have investigated the risk factors or outcomes associated with child developmental patterns, identified using the LCTM or its analogous models.^11,12,35^ In addition to these previous reports, we identified new patterns in 5 respective domains using ASQ-3 data across multiple time points from a large sample, which ensured consistency in assessment and reliability. To our knowledge, our study is the first to clarify the association between HDP subtypes and domain-specific trajectories. While this longitudinal outcome can be interpreted by linear mixed-effect models as well, their capacities to capture patterns is limited, precluding the comparisons of risk patterns, which is critical in public health contexts. They are more concise and robust to capture an overall trend, although no material difference was longitudinally observed in the current topic.

We found an association between exposure to HDP, especially the preeclampsia subtype, during the fetal stage and a delay pattern in the problem solving child developmental domain. In mouse models, reduced angiogenesis was reported in the brain cortex of offspring from preeclampsia models,^36^ while in humans, reduced fetal brain T2* (tissue oxygenation) and volume have been reported in pregnant women with preeclampsia.^37^ Multiple domains are probably affected by preeclampsia-induced pathological changes occurring during the fetal period, resulting in an overall impact on fetal brain development.

Children exposed to early-onset preeclampsia, but not late-onset preeclampsia, were more likely to have RRs greater than 1 for the adverse patterns. Both early-onset preeclampsia and late-onset preeclampsia result from placental syncytiotrophoblast stress; however, early-onset preeclampsia is more strongly associated with spiral artery defects, while late-onset preeclampsia might be secondary to intervillous malperfusions due to maternal cardiovascular system inabilities.^38^ On the genetic level, dysregulated genes in the placenta are functionally distinct in early-onset preeclampsia and late-onset preeclampsia subtypes.^39^ These findings support the idea that early-onset preeclampsia and late-onset preeclampsia affect children’s outcomes through different pathways.

In the term-born population, early-onset preeclampsia was not associated with the catch-up pattern, which may be attributable to the effects of premature birth in very young children. The significant risk of the delay pattern disappeared in most domains as well. A study by van Wassenaer et al^40^ followed up on children born after expectant management in pregnancies with fetal growth restriction and preeclampsia and found no behavioral problems. This suggests that delaying delivery until term helps the offspring maintain a normal developmental pattern. Our findings support the American College of Obstetricians and Gynecologists practice bulletins^41^ that observation until term delivery is recommended in women with preeclampsia without severe features. Preeclampsia-exposed children still have a risk of delay in the problem solving domain. Similar risks have been reported for autism, attention-deficit/hyperactivity disorder, epilepsy, and intellectual disability in children born at term who were exposed to preeclampsia.^42^ Notably, children born to mothers with preeclampsia exhibit increased resting-state functional connectivity between the amygdala and frontal poles—brain regions implicated in goal-directed behavior.^43,44^ Thus, their progression in problem solving warrants close monitoring, irrespective of whether they are born at term.

Preterm birth modified the association between early-onset preeclampsia and adverse developmental patterns in gross motor and problem solving domains. Approximately 40% to 70% preterm births are complicated with chorioamnionitis, leading to an infectious or microbial activation of the innate immune system, including interferon regulatory factor 1, which plays an important role in inflammation regulation in the central nervous system.^45,46,47^ Moreover, preterm neonates often present with immature swallowing function, characterized by unrefined upper and lower esophageal sphincter kinetics. For such infants, tube-feeding is a long-accepted strategy, but it has been reported to impair neuromotor coordination in their infancy,^48,49^ thereby increasing risk of persistent difficulty in motors and problem solving.

In the sensitivity analysis conducted in the complete-case population, some results significant in the main analysis lost their significance. A possible reason is that the population decreased by 2249 mother-child pairs, which reduced statistical power. Additionally, multiple imputation was conducted under the hypothesis that the covariates were missing at random. However, missing data may be related to HDP subtypes or developmental patterns, ie, not missing completely at random.

### Strengths and Limitations

This study has several strengths. First, it was based on a large-scale cohort study of more than 10 000 mother-child pairs, which ensures low risk of overfitting in the developmental patterns we generated while maintaining high statistical power. Second, we used LCTM to degrade ASQ-3 data. The LCTM identifies patterns without a detailed definition of cut off values or time points. This enables the inclusion of participants with acceptable missing numbers of ASQ-3 responses, avoiding losses in the exclusion step for ASQ-3 missing responses. Third, the effects of preterm birth on development were ruled out. Fourth, a series of sensitivity analyses were conducted to ensure the robustness of the results.

Additionally, this study has some limitations. In our determination of child developmental patterns, child behaviors after birth, such as education and nutrition, as well as environmental factors, were not considered. Neither were the patterns validated through external cohorts or internally split test set. We evaluated under the premise that developmental domains are independent, although evidence indicates that cognitive and motor development are closely associated with each other.^50^ Further studies are warranted to determine the possible developmental patterns across domains. Development measurements were included until age 48 months, which only represented early childhood. The TMM BirThree Cohort Study will continue to collect developmental data, aiming to generalize developmental patterns that extend to adolescence and adulthood. We excluded 7866 mother-child pairs with fewer than 2 responses to ASQ-3. Some of the baseline characteristics differed between these excluded and included populations, which may have led to selection bias. The TMM BirThree Cohort Study is a regional cohort study conducted in Japan. Therefore, our results should be validated in other regions and racial and ethnic groups.

## Conclusions

In this cohort study, fetal exposure to early-onset preeclampsia was associated with a higher risk of a delay pattern in child development in the problem solving domain. The findings were modified by preterm birth. These findings suggest that children exposed to preeclampsia in utero require monitoring in their progression in gross motor and problem solving function, especially if they were born preterm.

## Notes

Supplement 1. **eFigure 1.** Participant Selection Flow of the Study **eFigure 2.** ASQ-3 Score Distribution in Three Developmental Patterns in Five Domains **eMethods.** Imputation Methods for Missing Covariates **eTable 1. **Baseline Characteristics of Mother-Child Pairs in the HDP Subtypes **eTable 2. **Baseline Characteristics of Mother-Child Pairs Before Imputation According to HDP Prevalence **eTable 3. **Baseline Characteristics of the Participant Mother-Child Pairs Before Imputation in the HDP Subtypes **eTable 4. **Developmental Pattern Distribution Across HDP and Subtypes, in Term Birth and Preterm Birth Population **eTable 5.** Model Adequacy Assessments in the Latent Class Trajectory Model **eTable 6.** Congenital Abnormalities Excluded in the Sensitivity Analysis **eTable 7.** Number of ASQ-3 Responses in the Analysis Population **eTable 8.** Relative Risks in Child Developmental Patterns Across HDP and Subtypes in Complete Cases and After Excluding Congenital Abnormalities and Differences in Longitudinal Trends in Child Development by HDP Subtypes, Estimated by Linear Mix Model **eTable 9.** Baseline Characteristics Among Participants Included and Excluded by ASQ-3 Response

Supplement 2.
Data Sharing Statement

## References

[1] Jiang L, Tang K, Magee LA, . A global view of hypertensive disorders and diabetes mellitus during pregnancy. Nat Rev Endocrinol. 2022;18(12):760-775. doi:10.1038/s41574-022-00734-y 36109676 PMC9483536

[2] Huang C, Wei K, Lee PMY, Qin G, Yu Y, Li J. Maternal hypertensive disorder of pregnancy and mortality in offspring from birth to young adulthood: national population based cohort study. BMJ. 2022;379:e072157. doi:10.1136/bmj-2022-072157 36261141 PMC9580246

[3] Malhamé I, Nerenberg K, McLaughlin K, Grandi SM, Daskalopoulou SS, Metcalfe A. Hypertensive disorders and cardiovascular severe maternal morbidity in the US, 2015-2019. JAMA Netw Open. 2024;7(10):e2436478. doi:10.1001/jamanetworkopen.2024.36478 39361284 PMC11581633

[4] Pittara T, Vyrides A, Lamnisos D, Giannakou K. Pre-eclampsia and long-term health outcomes for mother and infant: an umbrella review. BJOG. 2021;128(9):1421-1430. 33638891 10.1111/1471-0528.16683

[5] Bellman M, Byrne O, Sege R. Developmental assessment of children. BMJ. 2013;346:e8687. doi:10.1136/bmj.e8687 23321410

[6] Harstad E, Hanson E, Brewster SJ, . Persistence of autism spectrum disorder from early childhood through school age. JAMA Pediatr. 2023;177(11):1197-1205. doi:10.1001/jamapediatrics.2023.4003 37782510 PMC10546296

[7] Hoeksma MR, Kemner C, Verbaten MN, van Engeland H. Processing capacity in children and adolescents with pervasive developmental disorders. J Autism Dev Disord. 2004;34(3):341-354. doi:10.1023/B:JADD.0000029555.98493.36 15264501

[8] Chen G, Ishikuro M, Ohseto H, . Hypertensive disorders of pregnancy, neonatal outcomes and offspring developmental delay in Japan: the Tohoku Medical Megabank Project Birth and Three-Generation Cohort Study. Acta Obstet Gynecol Scand. 2024;103(6):1192-1200. doi:10.1111/aogs.14820 38454539 PMC11103128

[9] Melough MM, Li M, Hamra G, ; program collaborators for Environmental influences on Child Health Outcomes. Greater gestational vitamin D Status is associated with reduced childhood behavioral problems in the environmental influences on child health outcomes program. J Nutr. 2023;153(5):1502-1511. doi:10.1016/j.tjnut.2023.03.005 37147034 PMC10367223

[10] Nagin D. Group-Based Modeling of Development. Harvard University Press; 2005. doi:10.4159/9780674041318

[11] Li QQ, Huang J, Cai D, . Prenatal exposure to legacy and alternative per- and polyfluoroalkyl substances and neuropsychological development trajectories over the first 3 years of life. Environ Sci Technol. 2023;57(9):3746-3757. doi:10.1021/acs.est.2c07807 36800558

[12] Black M, Adjei NK, Strong M, Barnes A, Jordan H, Taylor-Robinson D. Trajectories of child cognitive and socioemotional development and associations with adolescent health in the UK millennium cohort study. J Pediatr. 2023;263:113611. doi:10.1016/j.jpeds.2023.113611 37468036

[13] Luby JL, Belden A, Harms MP, Tillman R, Barch DM. Preschool is a sensitive period for the influence of maternal support on the trajectory of hippocampal development. Proc Natl Acad Sci U S A. 2016;113(20):5742-5747. doi:10.1073/pnas.1601443113 27114522 PMC4878487

[14] Mikami M, Hirota T, Adachi M, . Trajectories of emotional and behavioral problems in school-age children with coordination difficulties and their relationships to ASD/ADHD traits. Res Dev Disabil. 2023;133:104394. doi:10.1016/j.ridd.2022.104394 36543036

[15] Nagata A, Masumoto T, Nishigori H, Nakagawa T, Otani S, Kurozawa Y; Japan Environment and Children’s Study Group. Neurodevelopmental outcomes among offspring exposed to corticosteroid and B_2_-adrenergic agonists in utero. JAMA Netw Open. 2023;6(10):e2339347. doi:10.1001/jamanetworkopen.2023.39347 37874567 PMC10599123

[16] Zhou Y, Zhang L, Wang P, . Prenatal organophosphate esters exposure and neurodevelopment trajectory in infancy: evidence from the Shanghai Maternal-Child Pairs Cohort. Sci Total Environ. 2024;927:172366. doi:10.1016/j.scitotenv.2024.172366 38614325

[17] Zhu Z, Chang S, Cheng Y, . Early life cognitive development trajectories and intelligence quotient in middle childhood and early adolescence in rural western China. Sci Rep. 2019;9(1):18315. doi:10.1038/s41598-019-54755-1 31797987 PMC6892923

[18] Turesky TK, Escalante ES, Loh M, Gaab N. Longitudinal trajectories of brain development from infancy to school age and their relationship with literacy development. Proc Natl Acad Sci U S A. 2025;122(24):e2414598122. doi:10.1073/pnas.2414598122 40493188 PMC12184337

[19] Corrêa RRM, Gilio DB, Cavellani CL, . Placental morphometrical and histopathology changes in the different clinical presentations of hypertensive syndromes in pregnancy. Arch Gynecol Obstet. 2008;277(3):201-206. doi:10.1007/s00404-007-0452-z 17786461

[20] Maloney KF, Heller D, Baergen RN. Types of maternal hypertensive disease and their association with pathologic lesions and clinical factors. Fetal Pediatr Pathol. 2012;31(5):319-323. doi:10.3109/15513815.2012.659391 22432966

[21] Staff AC. The two-stage placental model of preeclampsia: an update. J Reprod Immunol. 2019;134-135:1-10. doi:10.1016/j.jri.2019.07.004 31301487

[22] Kuriyama S, Yaegashi N, Nagami F, . The Tohoku Medical Megabank Project: design and mission. J Epidemiol. 2016;26(9):493-511. doi:10.2188/jea.JE20150268 27374138 PMC5008970

[23] Sugawara J, Ishikuro M, Obara T, . Maternal baseline characteristics and perinatal outcomes: the Tohoku Medical Megabank Project Birth and Three-Generation Cohort Study. J Epidemiol. 2022;32(2):69-79. doi:10.2188/jea.JE20200338 33041318 PMC8761563

[24] Ishikuro M, Obara T, Osanai T, . Strategic methods for recruiting grandparents: the Tohoku Medical Megabank Birth and Three-Generation Cohort Study. Tohoku J Exp Med. 2018;246(2):97-105. doi:10.1620/tjem.246.97 30333380

[25] Mizuno S, Wagata M, Nagaie S, . Development of phenotyping algorithms for hypertensive disorders of pregnancy (HDP) and their application in more than 22,000 pregnant women. Sci Rep. 2024;14(1):6292. doi:10.1038/s41598-024-55914-9 38491024 PMC10943000

[26] Squires J, Bricker D. ASQ-3 Ages & Stages Questionnaires/ASQ-3 User’s Guide: A Parent-Completed Child-Monitoring System. Brooks Publishing Co; 2009.

[27] Mezawa H, Aoki S, Nakayama SF, . Psychometric profile of the Ages and Stages Questionnaires, Japanese translation. Pediatr Int. 2019;61(11):1086-1095. doi:10.1111/ped.13990 31419360 PMC6899956

[28] Azur MJ, Stuart EA, Frangakis C, Leaf PJ. Multiple imputation by chained equations: what is it and how does it work? Int J Methods Psychiatr Res. 2011;20(1):40-49. doi:10.1002/mpr.329 21499542 PMC3074241

[29] van Buuren S, Groothuis-Oudshoorn K. MICE: multivariate imputation by chained equations in R. J Stat Softw. 2011;45(3):1-67. doi:10.18637/jss.v045.i03

[30] Lennon H, Kelly S, Sperrin M, . Framework to construct and interpret latent class trajectory modelling. BMJ Open. 2018;8(7):e020683. doi:10.1136/bmjopen-2017-020683 29982203 PMC6042544

[31] Proust-Lima C, Philipps V, Liquet B. Estimation of extended mixed models using latent classes and latent processes: the R package lcmm. J Stat Softw. 2017;78(2):1-56. doi:10.18637/jss.v078.i02

[32] Mitha A, Chen R, Razaz N, . Neurological development in children born moderately or late preterm: national cohort study. BMJ. 2024;384:e075630. doi:10.1136/bmj-2023-075630 38267070 PMC11957549

[33] The R Core Team. R: A Language and Environment for Statistical Computing. R Foundation for Statistical Computing; 2018.

[34] Cao J, Zhang S. Multiple comparison procedures. JAMA. 2014;312(5):543-544. doi:10.1001/jama.2014.9440 25096694

[35] McCormick BJJ, Caulfield LE, Richard SA, ; MAL-ED Network Investigators. Early life experiences and trajectories of cognitive development. Pediatrics. 2020;146(3):e20193660. doi:10.1542/peds.2019-3660 32817437 PMC7461241

[36] Troncoso F, Sandoval H, Ibañez B, . Reduced brain cortex angiogenesis in the offspring of the preeclampsia-like syndrome. Hypertension. 2023;80(12):2559-2571. doi:10.1161/HYPERTENSIONAHA.123.21756 37767691 PMC7618073

[37] Hall M, de Marvao A, Schweitzer R, . Preeclampsia associated differences in the placenta, fetal brain, and maternal heart can be demonstrated antenatally: An observational cohort study using MRI. Hypertension. 2024;81(4):836-847. doi:10.1161/HYPERTENSIONAHA.123.22442 38314606 PMC7615760

[38] Ortega MA, Fraile-Martínez O, García-Montero C, . The pivotal role of the placenta in Normal and pathological pregnancies: a focus on preeclampsia, fetal growth restriction, and maternal chronic venous disease. Cells. 2022;11(3):568. doi:10.3390/cells11030568 35159377 PMC8833914

[39] Ren Z, Gao Y, Gao Y, . Distinct placental molecular processes associated with early-onset and late-onset preeclampsia. Theranostics. 2021;11(10):5028-5044. doi:10.7150/thno.56141 33754042 PMC7978310

[40] van Wassenaer AG, Westera J, van Schie PEM, . Outcome at 4.5 years of children born after expectant management of early-onset hypertensive disorders of pregnancy. Am J Obstet Gynecol. 2011;204(6):510.e1-510.e9. doi:10.1016/j.ajog.2011.02.032 21459356

[41] American College of Obstetricians and Gynecologists. Gestational hypertension and preeclampsia: ACOG practice bulletin summary, Number 222. Obstet Gynecol. 2020;135(6):1492-1495. doi:10.1097/AOG.0000000000003892 32443077

[42] Sun BZ, Moster D, Harmon QE, Wilcox AJ. Association of preeclampsia in term births with neurodevelopmental disorders in offspring. JAMA Psychiatry. 2020;77(8):823-829. doi:10.1001/jamapsychiatry.2020.0306 32236510 PMC7113825

[43] Mak LE, Croy BA, Kay V, . Resting-state functional connectivity in children born from gestations complicated by preeclampsia: a pilot study cohort. Pregnancy Hypertens. 2018;12:23-28. doi:10.1016/j.preghy.2018.02.004 29674194

[44] Amodio DM, Frith CD. Meeting of minds: the medial frontal cortex and social cognition. Nat Rev Neurosci. 2006;7(4):268-277. doi:10.1038/nrn1884 16552413

[45] Yoon BH, Romero R, Moon JB, . Clinical significance of intra-amniotic inflammation in patients with preterm labor and intact membranes. Am J Obstet Gynecol. 2001;185(5):1130-1136. doi:10.1067/mob.2001.117680 11717646

[46] Phung J, Wang C, Reeders J, . Preterm labor with and without chorioamnionitis is associated with activation of myometrial inflammatory networks: a comprehensive transcriptomic analysis. Am J Obstet Gynecol. 2023;228(3):330.e1-330.e18. doi:10.1016/j.ajog.2022.08.036 36002050

[47] Yang X, Diaz V, Huang H. The role of interferon regulatory factor 1 in regulating microglial activation and retinal inflammation. Int J Mol Sci. 2022;23(23):14664. doi:10.3390/ijms232314664 36498991 PMC9739975

[48] Jadcherla SR, Shubert TR, Gulati IK, Jensen PS, Wei L, Shaker R. Upper and lower esophageal sphincter kinetics are modified during maturation: effect of pharyngeal stimulus in premature infants. Pediatr Res. 2015;77(1-1):99-106. doi:10.1038/pr.2014.147 25279989 PMC4268006

[49] Li XL, Liu Y, Liu M, Yang CY, Yang QZ. Early premature infant oral motor intervention improved oral feeding and prognosis by promoting neurodevelopment. Am J Perinatol. 2020;37(6):626-632. doi:10.1055/s-0039-1685448 31013539

[50] Davis EE, Pitchford NJ, Limback E. The interrelation between cognitive and motor development in typically developing children aged 4-11 years is underpinned by visual processing and fine manual control. Br J Psychol. 2011;102(3):569-584. doi:10.1111/j.2044-8295.2011.02018.x 21752007

