# Supplementary material for: Child Developmental Patterns by Age 4 Years Across Subtypes of Hypertensive Disorders of Pregnancy
Source: JAMA Netw Open. 2025 Nov 26;8(11):e2545719. doi: 10.1001/jamanetworkopen.2025.45719 (PMC12658654; doi:10.1001/jamanetworkopen.2025.45719)
Supplement: Supplement 1. — eFigure 1. Participant Selection Flow of the Study eFigure 2. ASQ-3 Score Distribution in Three Developmental Patterns in Five Domains eMethods. Imputation Methods for Missing Covariates eTable 1. Baseline Characteristics of Mother-Child Pairs in the HDP Subtypes eTable 2. Baseline Characteristics of Mother-Child Pairs Before Imputation According to HDP Prevalence eTable 3. Baseline Characteristics of the Participant Mother-Child Pairs Before Imputation in the HDP Subtypes eTable 4. Developmental Pattern Distribution Across HDP and Subtypes, in Term Birth and Preterm Birth Population eTable 5. Model Adequacy Assessments in the Latent Class Trajectory Model eTable 6. Congenital Abnormalities Excluded in the Sensitivity Analysis eTable 7. Number of ASQ-3 Responses in the Analysis Population eTable 8. Relative Risks in Child Developmental Patterns Across HDP and Subtypes in Complete Cases and After Excluding Congenital Abnormalities and Differences in Longitudinal Trends in Child Development by HDP Subtypes, Estimated by Linear Mix Model eTable 9. Baseline Characteristics Among Participants Included and Excluded by ASQ-3 Response [file jamanetwopen-e2545719-s001.pdf]

# Supplemental Online Content

Chen G, Ishikuro M, Ohseto H, et al. Child developmental patterns by age 4 years across subtypes of hypertensive disorders of pregnancy. *JAMA Netw Open*. 2025;8(11):e2545719. doi:10.1001/jamanetworkopen.2025.45719

**eFigure 1.** Participant Selection Flow of the Study
**eFigure 2.** ASQ-3 Score Distribution in Three Developmental Patterns in Five Domains
**eMethods.** Imputation Methods for Missing Covariates
**eTable 1.** Baseline Characteristics of Mother-Child Pairs in the HDP Subtypes
**eTable 2.** Baseline Characteristics of Mother-Child Pairs Before Imputation According to HDP Prevalence
**eTable 3.** Baseline Characteristics of the Participant Mother-Child Pairs Before Imputation in the HDP Subtypes
**eTable 4.** Developmental Pattern Distribution Across HDP and Subtypes, in Term Birth and Preterm Birth Population
**eTable 5.** Model Adequacy Assessments in the Latent Class Trajectory Model
**eTable 6.** Congenital Abnormalities Excluded in the Sensitivity Analysis
**eTable 7.** Number of ASQ-3 Responses in the Analysis Population
**eTable 8.** Relative Risks in Child Developmental Patterns Across HDP and Subtypes in Complete Cases and After Excluding Congenital Abnormalities and Differences in Longitudinal Trends in Child Development by HDP Subtypes, Estimated by Linear Mix Model
**eTable 9.** Baseline Characteristics Among Participants Included and Excluded by ASQ-3 Response

This supplemental material has been provided by the authors to give readers additional information about their work.

**eFigure 1. Participant selection flow of the study.**
14023 mother-child pairs were included in the main analysis. Abbreviations: HDP, hypertensive disorders of pregnancy; ASQ-3, Age and Stages Questionnaires, third edition; NA, not available.

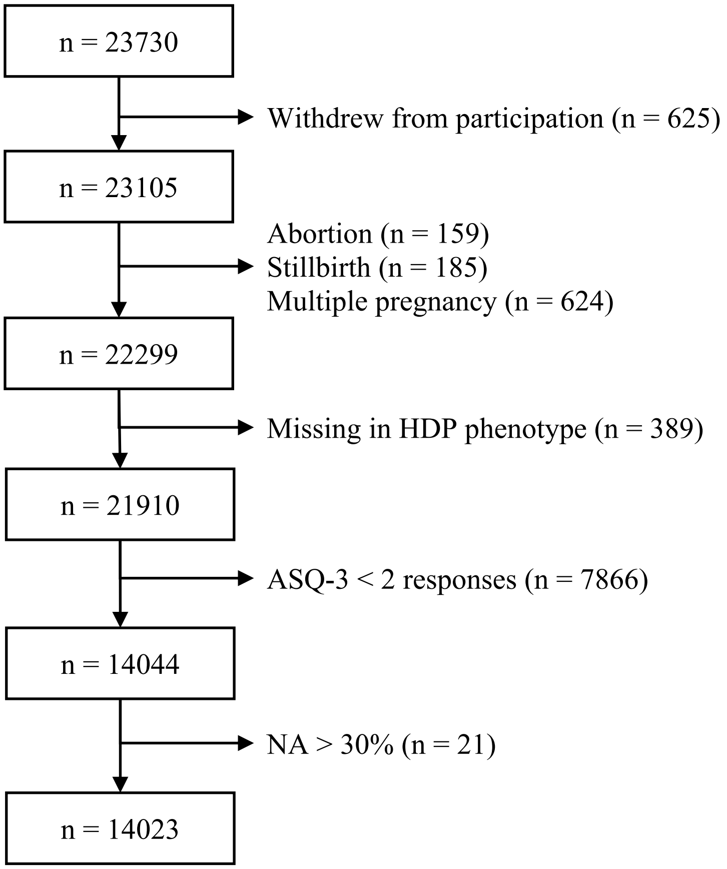


© 2025 Chen G et al. *JAMA Network Open*.

## eFigure 2. ASQ-3 score distribution in three developmental patterns in five domains.

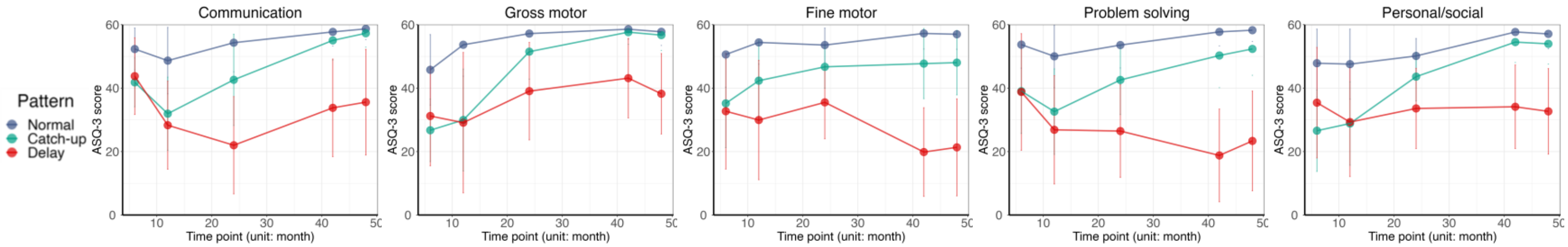


The mean and SDs of the participants' ASQ-3 scores were calculated by time point and domain and grouped by developmental patterns identified using LCTM. Dots represent the mean values at a given site, whereas vertical bars indicate SDs. Each color corresponds to a specific pattern: normal (blue), catch-up (green), or delay (red). From left to right, five developmental domains were presented: communication, gross motor, fine motor, problem solving, and personal/social. The trends in mean values were consistent with the prediction graph (Figure 1). ASQ-3, Ages and Stages Questionnaires, third edition; SD, standard deviation; LCTM, latent class trajectory model

© 2025 Chen G et al. *JAMA Network Open*.

## eMethods. Imputation Methods for Missing Covariates

According to the missing pattern of the covariates in the study population, we assumed they were missing at random (MAR). After excluding participants with > 30% variables missing (eFigure 1), we used multiple imputation with chained equations to impute the missing values, through the R package "mice" (version 3.14.0). All the variables in the substantive analysis were included in this process.

Parity, maternal tobacco use in pregnancy, and maternal alcohol use in pregnancy were imputed using the logistic regression model. Maternal educational attainments, family annual income, maternal folic acid intake in pregnancy were imputed using the polytomous logistic regression model. Maternal pre-pregnancy body max index (BMI) was imputed using predictive mean matching, then categorized into 3 levels. There was no missing in maternal age, gestational diabetes mellitus prevalence or child sex.

We imputed missing values for 20 imputations. For each imputed dataset, the chained equations algorithm was run for 5 iterations. Substantive analyses were conducted independently in 20 imputed datasets, then pooled under the Rubin's rule.

Baseline characteristics of participants before and after imputation according to HDP prevalence and HDP subtypes are shown in Table 1, eTables 1-4.

© 2025 Chen G et al. *JAMA Network Open*.

## eTable 1. Baseline characteristics of mother–child pairs in the HDP subtypes

| Characteristics | Mother-child pairs, No. (%) | | | | |
|---|---|---|---|---|---|
| | CH (n=328) | GH (n=552) | PE (n=349) | PE EO (n=84) | PE LO (n=265) |
| **Maternal age, year, mean (SD)** | 33.4 (5.1) | 32.8 (5.2) | 32.8 (5.2) | 32.1 (5.6) | 33.0 (5.1) |
| **Family income, JPY/year** | | | | | |
| < 4 000 000 | 120 (36.6) | 221 (40.0) | 143 (41.0) | 45 (53.6) | 98 (37.0) |
| ≥ 4 000 000, < 6 000 000 | 122 (37.2) | 173 (31.3) | 102 (29.2) | 22 (26.2) | 80 (30.2) |
| ≥ 6 000 000 | 86 (26.2) | 158 (28.6) | 104 (29.8) | 17 (20.2) | 87 (32.8) |
| **Maternal educational level** | | | | | |
| High school or lower | 130 (39.6) | 212 (38.4) | 141 (40.4) | 40 (47.6) | 101 (38.1) |
| Junior or vocational college | 125 (38.1) | 223 (40.4) | 129 (37.0) | 26 (31.0) | 103 (38.9) |
| University or higher | 73 (22.3) | 117 (21.2) | 79 (22.6) | 18 (21.4) | 61 (23.0) |
| **Maternal pre-pregnancy BMI[a], kg/m²** | | | | | |
| < 18.5 | 177 (54.0) | 363 (65.8) | 230 (65.9) | 51 (60.7) | 179 (67.5) |
| ≥ 18.5, < 25 | 15 (4.6) | 61 (11.1) | 36 (10.3) | 8 (9.5) | 28 (10.6) |
| ≥ 25 | 136 (41.5) | 128 (23.2) | 83 (23.8) | 25 (29.8) | 58 (21.9) |
| **Parity** | | | | | |
| Multipara | 158 (48.2) | 247 (44.7) | 140 (40.1) | 34 (40.5) | 106 (40.0) |
| **Gestational diabetes mellitus prevalence** | | | | | |
| Yes | 17 (5.2) | 17 (3.1) | 7 (2.0) | 3 (3.6) | 4 (1.5) |
| **Maternal tobacco use** | | | | | |
| Yes | 40 (12.2) | 87 (15.8) | 57 (16.3) | 16 (19.0) | 41 (15.5) |
| **Maternal alcohol use** | | | | | |
| Yes | 71 (21.6) | 116 (21.0) | 76 (21.8) | 19 (22.6) | 57 (21.5) |
| **Maternal folic acid intake** | | | | | |
| No during pregnancy | 125 (38.1) | 200 (36.2) | 122 (35.0) | 36 (42.9) | 86 (32.5) |
| Yes during pregnancy | 131 (39.9) | 249 (45.1) | 161 (46.1) | 39 (46.4) | 122 (46.0) |
| Yes before and during pregnancy | 72 (22.0) | 103 (18.7) | 66 (18.9) | 9 (10.7) | 57 (21.5) |
| **Preterm Birth** | | | | | |
| Yes | 26 (7.9) | 39 (7.1) | 47 (13.5) | 28 (33.3) | 19 (7.2) |
| **Child sex** | | | | | |
| Female | 144 (43.9) | 257 (46.6) | 180 (51.6) | 42 (50.0) | 138 (52.1) |

[a]BMI was calculated by dividing the pre-pregnancy weight (kg) by the square of height ($m^2$)
CH, chronic hypertension; GH, gestational hypertension; PE, preeclampsia; EO, early onset; LO, late onset; JPY, Japanese Yen; BMI, body mass index.

© 2025 Chen G et al. *JAMA Network Open*.

**eTable 2. Baseline characteristics of mother–child pairs before imputation according to HDP prevalence**

| Characteristics | Mother-child pairs, No. (%) | | | *P*-value |
|---|---|---|---|---|
| | Total (n=14023) | HDP-unaffected (n=12617) | HDP-affected (n=1406) | |
| **Maternal age, year, mean (SD)** | 32.5 (4.8) | 32.5 (4.8) | 33.1 (5.2) | <0.001 |
| **Family income, JPY/year** | | | | 0.001 |
| < 4 000 000 | 4608 (32.9) | 4102 (32.5) | 506 (36.0) | |
| ≥ 4 000 000, < 6 000 000 | 4410 (31.4) | 3978 (31.5) | 432 (30.7) | |
| ≥ 6 000 000 | 4254 (30.3) | 3880 (30.8) | 374 (26.6) | |
| **Maternal educational level** | | | | <0.001 |
| High school or lower | 4096 (29.2) | 3602 (28.5) | 494 (35.1) | |
| Junior or vocational college | 4909 (35.0) | 4425 (35.1) | 484 (34.4) | |
| University or higher | 3643 (26.0) | 3359 (26.6) | 284 (20.2) | |
| **Maternal pre-pregnancy BMI[a], kg/m$^2$** | | | | <0.001 |
| < 18.5 | 10280 (73.3) | 9433 (74.8) | 847 (60.2) | |
| ≥ 18.5, < 25 | 1818 (13.0) | 1691 (13.4) | 127 (9.0) | |
| ≥ 25 | 1704 (12.2) | 1291 (10.2) | 413 (29.4) | |
| **Parity** | | | | <0.001 |
| Multipara | 7391 (52.7) | 6764 (53.6) | 627 (44.6) | |
| **Gestational diabetes mellitus prevalence** | | | | <0.001 |
| Yes | 328 (2.3) | 275 (2.2) | 53 (3.8) | |
| **Maternal tobacco use** | | | | 0.309 |
| Yes | 1876 (13.4) | 1675 (13.3) | 201 (14.3) | |
| **Maternal alcohol use** | | | | 0.178 |
| Yes | 3028 (21.6) | 2733 (21.7) | 295 (21.0) | |
| **Maternal folic acid intake** | | | | 0.529 |
| No during pregnancy | 5238 (37.4) | 4722 (37.4) | 516 (36.7) | |
| Yes during pregnancy | 6160 (43.9) | 5551 (44.0) | 609 (43.3) | |
| Yes before and during pregnancy | 2490 (17.8) | 2226 (17.6) | 264 (18.8) | |
| **Preterm Birth** | | | | <0.001 |
| Yes | 715 (5.1) | 580 (4.6) | 135 (9.6) | |
| **Child sex** | | | | 0.666 |
| Female | 6754 (48.2) | 6085 (48.2) | 669 (47.6) | |

[a]BMI was calculated by dividing the pre-pregnancy weight (kg) by the square of height (m$^2$)
HDP, hypertensive disorders of pregnancy; JPY, Japanese Yen; BMI, body mass index; NA, not applicable.

© 2025 Chen G et al. *JAMA Network Open*.

**eTable 3. Baseline characteristics of the participant mother–child pairs before imputation in the HDP subtypes**

| Characteristics | Mother-child pairs, No. (%) | | | | |
|---|---|---|---|---|---|
| | CH (n=328) | GH (n=552) | PE (n=349) | PE EO (n=84) | PE LO (n=265) |
| **Maternal age, year, mean (SD)** | 33.4 (5.1) | 32.8 (5.2) | 32.8 (5.2) | 32.1 (5.6) | 33.0 (5.1) |
| **Family income, JPY/year** | | | | | |
| < 4 000 000 | 109 (33.2) | 198 (35.9) | 136 (39.0) | 40 (47.6) | 96 (36.2) |
| ≥ 4 000 000, < 6 000 000 | 118 (36.0) | 163 (29.5) | 98 (28.1) | 19 (22.6) | 79 (29.8) |
| ≥ 6 000 000 | 82 (25.0) | 146 (26.4) | 93 (26.6) | 16 (19.0) | 77 (29.1) |
| **Maternal educational level** | | | | | |
| High school or lower | 112 (34.1) | 182 (33.0) | 129 (37.0) | 35 (41.7) | 94 (35.5) |
| Junior or vocational college | 118 (36.0) | 198 (35.9) | 116 (33.2) | 23 (27.4) | 93 (35.1) |
| University or higher | 68 (20.7) | 104 (18.8) | 73 (20.9) | 16 (19.0) | 57 (21.5) |
| **Maternal pre-pregnancy BMI[a], kg/m$^2$** | | | | | |
| < 18.5 | 176 (53.7) | 361 (65.4) | 225 (64.5) | 48 (57.1) | 177 (66.8) |
| ≥ 18.5, < 25 | 15 (4.6) | 60 (10.9) | 36 (10.3) | 8 (9.5) | 28 (10.6) |
| ≥ 25 | 133 (40.5) | 128 (23.2) | 81 (23.2) | 25 (29.8) | 56 (21.1) |
| **Parity** | | | | | |
| Multipara | 158 (48.2) | 247 (44.7) | 140 (40.1) | 34 (40.5) | 106 (40.0) |
| **Gestational diabetes mellitus prevalence** | | | | | |
| Yes | 17 (5.2) | 17 (3.1) | 7 (2.0) | 3 (3.6) | 4 (1.5) |
| **Maternal tobacco use** | | | | | |
| Yes | 40 (12.2) | 86 (15.6) | 56 (16.0) | 16 (19.0) | 40 (15.1) |
| **Maternal alcohol use** | | | | | |
| Yes | 70 (21.3) | 115 (20.8) | 76 (21.8) | 19 (22.6) | 57 (21.5) |
| **Maternal folic acid intake** | | | | | |
| No during pregnancy | 123 (37.5) | 197 (35.7) | 121 (34.7) | 35 (41.7) | 86 (32.5) |
| Yes during pregnancy | 131 (39.9) | 247 (44.7) | 159 (45.6) | 39 (46.4) | 120 (45.3) |
| Yes before and during pregnancy | 72 (22.0) | 100 (18.1) | 64 (18.3) | 9 (10.7) | 55 (20.8) |
| **Preterm Birth** | | | | | |
| Yes | 26 (7.9) | 39 (7.1) | 47 (13.5) | 28 (33.3) | 19 (7.2) |
| **Child sex** | | | | | |
| Female | 144 (43.9) | 257 (46.6) | 180 (51.6) | 42 (50.0) | 138 (52.1) |

[a]BMI was calculated by dividing the pre-pregnancy weight (kg) by the square of height (m$^2$).
CH, chronic hypertension; GH, gestational hypertension; PE, preeclampsia; EO, early onset; LO, late onset; JPY, Japanese Yen; BMI, body mass index.

© 2025 Chen G et al. *JAMA Network Open*.

## eTable 4-1. Developmental Pattern Distribution across HDP and Subtypes, in Term Birth Population

| Domain | Pattern | Mother-child pairs, No. (%) | | | | | | |
|---|---|---|---|---|---|---|---|---|
| | | Total (n=13307) | HDP-unaffected (n=12037) | HDP-affected (n=1270) | HDP subtypes | | | |
| | | | | | GH (n=513) | PE (n=302) | PE EO (n=56) | PE LO (n=246) |
| **Communication** | Normal | 8920 (67.0) | 8086 (67.2) | 834 (65.7) | 343 (66.9) | 194 (64.2) | 30 (53.6) | 164 (66.7) |
| | Catch-up | 3269 (24.6) | 2960 (24.6) | 309 (24.3) | 126 (24.6) | 76 (25.2) | 18 (32.1) | 58 (23.6) |
| | Delay | 1118 (8.4) | 991 (8.2) | 127 (10.0) | 44 (8.6) | 32 (10.6) | 8 (14.3) | 24 (9.8) |
| **Gross motor** | Normal | 9745 (73.2) | 8836 (73.4) | 909 (71.6) | 373 (72.7) | 213 (70.5) | 40 (71.4) | 173 (70.3) |
| | Catch-up | 2420 (18.2) | 2193 (18.2) | 227 (17.9) | 88 (17.2) | 58 (19.2) | 12 (21.4) | 46 (18.7) |
| | Delay | 1141 (8.6) | 1007 (8.4) | 134 (10.6) | 52 (10.1) | 31 (10.3) | 4 (7.1) | 27 (11.0) |
| **Fine motor** | Normal | 9479 (71.2) | 8621 (71.6) | 858 (67.6) | 342 (66.7) | 204 (67.5) | 38 (67.9) | 166 (67.5) |
| | Catch-up | 3467 (26.1) | 3094 (25.7) | 373 (29.4) | 159 (31.0) | 87 (28.8) | 16 (28.6) | 71 (28.9) |
| | Delay | 357 (2.7) | 318 (2.6) | 39 (3.1) | 12 (2.3) | 11 (3.6) | 2 (3.6) | 9 (3.7) |
| **Problem solving** | Normal | 10120 (76.1) | 9192 (76.4) | 928 (73.1) | 381 (74.3) | 218 (72.2) | 38 (67.9) | 180 (73.2) |
| | Catch-up | 2779 (20.9) | 2484 (20.6) | 295 (23.2) | 117 (22.8) | 66 (21.9) | 14 (25.0) | 52 (21.1) |
| | Delay | 394 (3.0) | 348 (2.9) | 46 (3.6) | 15 (2.9) | 17 (5.6) | 4 (7.1) | 13 (5.3) |
| **Personal/social** | Normal | 9508 (71.5) | 8620 (71.6) | 888 (69.9) | 361 (70.4) | 212 (70.2) | 40 (71.4) | 172 (69.9) |
| | Catch-up | 2931 (22.0) | 2643 (22.0) | 288 (22.7) | 117 (22.8) | 65 (21.5) | 13 (23.2) | 52 (21.1) |
| | Delay | 858 (6.4) | 764 (6.3) | 94 (7.4) | 35 (6.8) | 25 (8.3) | 3 (5.4) | 22 (8.9) |

HDP, hypertensive disorders of pregnancy; GH, gestational hypertension; PE, preeclampsia; EO, early onset; LO late onset.

© 2025 Chen G et al. *JAMA Network Open*.

## eTable 4-2. Developmental Pattern Distribution across HDP and Subtypes, in Preterm Birth Population

| Domain | Pattern | Mother-child pairs, No. (%) | | | | | | |
|---|---|---|---|---|---|---|---|---|
| | | Total (n=715) | HDP-unaffected (n=580) | HDP-affected (n=135) | HDP subtypes | | | |
| | | | | | GH (n=39) | PE (n=47) | PE EO (n=28) | PE LO (n=19) |
| **Communication** | Normal | 352 (49.2) | 294 (50.7) | 58 (43.0) | 18 (46.2) | 19 (40.4) | 12 (42.9) | 7 (36.8) |
| | Catch-up | 248 (34.7) | 198 (34.1) | 50 (37.0) | 12 (30.8) | 18 (38.3) | 10 (35.7) | 8 (42.1) |
| | Delay | 115 (16.1) | 88 (15.2) | 27 (20.0) | 9 (23.1) | 10 (21.3) | 6 (21.4) | 4 (21.1) |
| **Gross motor** | Normal | 350 (49.0) | 297 (51.2) | 53 (39.3) | 15 (38.5) | 14 (29.8) | 6 (21.4) | 8 (42.1) |
| | Catch-up | 230 (32.2) | 184 (31.7) | 46 (34.1) | 14 (35.9) | 16 (34.0) | 11 (39.3) | 5 (26.3) |
| | Delay | 135 (18.9) | 99 (17.1) | 36 (26.7) | 10 (25.6) | 17 (36.2) | 11 (39.3) | 6 (31.6) |
| **Fine motor** | Normal | 355 (49.7) | 29 (5.0) | 65 (48.1) | 19 (48.7) | 22 (46.8) | 11 (39.3) | 11 (57.9) |
| | Catch-up | 296 (41.4) | 240 (41.4) | 56 (41.5) | 18 (46.2) | 18 (38.3) | 12 (42.9) | 6 (31.6) |
| | Delay | 64 (9.0) | 50 (8.6) | 14 (10.4) | 2 (5.1) | 7 (14.9) | 5 (17.9) | 2 (10.5) |
| **Problem solving** | Normal | 386 (54.0) | 327 (56.4) | 59 (43.7) | 17 (43.6) | 19 (40.4) | 8 (28.6) | 11 (57.9) |
| | Catch-up | 272 (38.0) | 207 (35.7) | 65 (48.1) | 19 (48.7) | 23 (48.9) | 16 (57.1) | 7 (36.8) |
| | Delay | 56 (7.8) | 45 (7.8) | 11 (8.1) | 3 (7.7) | 5 (10.6) | 4 (14.3) | 1 (5.3) |
| **Personal/social** | Normal | 329 (46.0) | 277 (47.8) | 52 (38.5) | 14 (35.9) | 15 (31.9) | 6 (21.4) | 9 (47.4) |
| | Catch-up | 304 (42.5) | 243 (41.9) | 61 (45.2) | 19 (48.7) | 25 (53.2) | 17 (60.7) | 8 (42.1) |
| | Delay | 82 (11.5) | 60 (10.3) | 22 (16.3) | 6 (15.4) | 7 (14.9) | 5 (17.9) | 2 (10.5) |

HDP, hypertensive disorders of pregnancy; GH, gestational hypertension; PE, preeclampsia; EO, early onset; LO late onset.

© 2025 Chen G et al. *JAMA Network Open*.

## eTable 5. Model adequacy assessments in the latent class trajectory model

| n | Fixed effect | BIC | APPA for every class | | | | | | | OCC for every class | | | | | | |
|---|---|---|---|---|---|---|---|---|---|---|---|---|---|---|---|---|
| | | | 1 | 2 | 3 | 4 | 5 | 6 | 7 | 1 | 2 | 3 | 4 | 5 | 6 | 7 |
| **Communication** | | | | | | | | | | | | | | | | |
| 2 | linear | 147618.6 | 0.92 | 0.99 | | | | | | 178.7 | 4.737 | | | | | |
| 3 | linear | 145346.2 | 0.87 | 0.88 | 0.73 | | | | | 52.24 | 4.243 | 7.566 | | | | |
| 4 | linear | 145077.5 | 0.88 | 0.69 | 0.86 | 0.78 | | | | 239.9 | 6.223 | 4.258 | 28.47 | | | |
| 5 | linear | 145119.1 | 0.87 | NA | 0.47 | 0.63 | 0.76 | | | 213.4 | NA | 1.98 | 4.689 | 23.21 | | |
| 6 | linear | 145138.5 | 0.84 | 0.61 | 0.7 | NA | 0.57 | 0.62 | | 245.3 | 4.478 | 47.83 | NA | 2.231 | 14.3 | |
| 7 | linear | 145075.4 | 0.83 | 0.66 | 0.7 | 0.72 | NA | 0.56 | 0.7 | 296 | 5.201 | 78.44 | 89.31 | NA | 2.136 | 36.55 |
| 2 | quadratic | 147625 | 0.92 | 0.99 | | | | | | 179.8 | 4.711 | | | | | |
| 3 | quadratic | 145354.7 | 0.87 | 0.88 | 0.73 | | | | | 53.25 | 4.207 | 7.684 | | | | |
| 4 | quadratic | 145081.6 | 0.87 | 0.69 | 0.86 | 0.79 | | | | 228.7 | 6.215 | 4.257 | 28.92 | | | |
| 5 | quadratic | 145080.3 | 0.85 | 0.68 | 0.86 | 0.72 | 0.68 | | | 324.2 | 5.871 | 4.278 | 63.92 | 21.46 | | |
| 6 | quadratic | 145149.9 | 0.84 | 0.7 | 0.58 | NA | 0.48 | 0.62 | | 243.9 | 47.69 | 4.202 | NA | 2.037 | 13.16 | |
| 7 | quadratic | 145148.4 | 0.84 | 0.7 | 0.62 | 0.61 | NA | 0.6 | 0.59 | 263.4 | 59.75 | 37.5 | 4.913 | NA | 2.283 | 16.68 |
| 2 | cubic | 147634.4 | 0.92 | 0.99 | | | | | | 178.3 | 4.724 | | | | | |
| 3 | cubic | 145461.2 | 0.87 | 0.89 | 0.75 | | | | | 71.79 | 4.227 | 8.46 | | | | |
| 4 | cubic | 145237.2 | 0.86 | 0.79 | 0.87 | 0.72 | | | | 226.3 | 37.89 | 3.988 | 7.077 | | | |
| 5 | cubic | 145112.5 | 0.84 | 0.64 | 0.85 | 0.7 | 0.6 | | | 245.4 | 5.416 | 4.154 | 45.01 | 14.16 | | |
| 6 | cubic | 145294.4 | 0.85 | 0.46 | 0.43 | 0.6 | 0.72 | 0.52 | | 217.2 | 4.571 | 1.797 | 2.979 | 37.54 | 7.953 | |
| 7 | cubic | 145252.6 | 0.85 | 0.69 | 0.55 | NA | 0.46 | 0.56 | 0.34 | 233.4 | 39.86 | 15.05 | NA | 3.58 | 2.154 | 4.622 |
| **Gross motor** | | | | | | | | | | | | | | | | |
| 2 | linear | 146470.7 | 0.9 | 0.98 | | | | | | 103.7 | 3.833 | | | | | |
| 3 | linear | 143839.2 | 0.87 | 0.9 | 0.74 | | | | | 50.13 | 4.315 | 11.55 | | | | |
| 4 | linear | 143310.7 | 0.9 | 0.89 | 0.69 | 0.81 | | | | 541.5 | 4.392 | 9.109 | 27.1 | | | |
| 5 | linear | 143339.4 | 0.89 | NA | 0.81 | 0.62 | 0.8 | | | 426.2 | NA | 2.943 | 8.388 | 25.42 | | |
| 6 | linear | 143393 | 0.88 | NA | 0.32 | 0.59 | 0.43 | 0.75 | | 330 | NA | 2.646 | 8.016 | 1.881 | 19.88 | |
| 7 | linear | 143428.5 | 0.88 | NA | 0.38 | 0.59 | NA | 0.29 | 0.75 | 342.9 | NA | 1.82 | 7.561 | NA | 2.627 | 20.43 |
| 2 | quadratic | 146479.8 | 0.9 | 0.98 | | | | | | 103.7 | 3.831 | | | | | |
| 3 | quadratic | 143851.3 | 0.87 | 0.9 | 0.74 | | | | | 51.83 | 4.288 | 11.65 | | | | |
| 4 | quadratic | 143319.2 | 0.9 | 0.89 | 0.69 | 0.81 | | | | 518.9 | 4.393 | 9.104 | 27.17 | | | |
| 5 | quadratic | 143397.9 | 0.9 | NA | 0.61 | 0.47 | 0.76 | | | 337.8 | NA | 6.324 | 1.85 | 18.68 | | |
| 6 | quadratic | 143464 | 0.86 | 0.58 | 0.31 | NA | 0.39 | 0.74 | | 198 | 6.883 | 2.117 | NA | 1.94 | 17.21 | |
| 7 | quadratic | 143458.1 | 0.79 | 0.66 | 0.53 | NA | NA | 0.32 | 0.52 | 177.3 | 24.78 | 5.118 | NA | NA | 1.714 | 8.743 |
| 2 | cubic | 145793.1 | 0.88 | 0.93 | | | | | | 24.66 | 3.571 | | | | | |
| 3 | cubic | 143940.1 | 0.87 | 0.9 | 0.78 | | | | | 69.54 | 4.093 | 12.29 | | | | |
| 4 | cubic | 143341.5 | 0.89 | 0.69 | 0.89 | 0.81 | | | | 372.7 | 9.112 | 4.397 | 26.63 | | | |

© 2025 Chen G et al. *JAMA Network Open*.

| n | Fixed effect | BIC | APPA for every class | | | | | | | OCC for every class | | | | | | |
|---|---|---|---|---|---|---|---|---|---|---|---|---|---|---|---|---|
| | | | 1 | 2 | 3 | 4 | 5 | 6 | 7 | 1 | 2 | 3 | 4 | 5 | 6 | 7 |
| **Gross motor** | | | | | | | | | | | | | | | | |
| 5 | cubic | 143424.5 | 0.89 | NA | 0.5 | 0.61 | 0.76 | | | 273.3 | NA | 1.906 | 6.407 | 18.63 | | |
| 6 | cubic | 143417.7 | 0.9 | 0.35 | 0.54 | NA | 0.46 | 0.74 | | 329.9 | 3.596 | 8.458 | NA | 1.855 | 18.78 | |
| 7 | cubic | 143451.1 | 0.88 | 0.64 | 0.39 | NA | 0.31 | 0.34 | 0.71 | 334.7 | 11.97 | 3.526 | NA | 1.728 | 1.99 | 21.36 |
| **Fine motor** | | | | | | | | | | | | | | | | |
| 2 | linear | 148776 | 0.87 | 0.9 | | | | | | 17.09 | 3.414 | | | | | |
| 3 | linear | 147718.5 | 0.86 | 0.88 | 0.81 | | | | | 146.5 | 3.783 | 9.667 | | | | |
| 4 | linear | 147323.7 | 0.87 | 0.64 | 0.88 | 0.78 | | | | 300.4 | 9.804 | 3.835 | 16.73 | | | |
| 5 | linear | 147362.4 | 0.87 | 0.59 | NA | 0.51 | 0.75 | | | 291.6 | 7.868 | NA | 1.816 | 13.6 | | |
| 6 | linear | 147361.3 | 0.83 | 0.45 | 0.57 | NA | 0.57 | 0.63 | | 210.8 | 9.479 | 1.917 | NA | 7.641 | 14.18 | |
| 7 | linear | 147578.2 | 0.88 | NA | 0.67 | NA | 0.26 | 0.23 | 0.56 | 313.1 | NA | 11.71 | NA | 1.631 | 1.506 | 6.697 |
| 2 | quadratic | 148786.9 | 0.87 | 0.9 | | | | | | 17.13 | 3.409 | | | | | |
| 3 | quadratic | 147727.3 | 0.86 | 0.88 | 0.81 | | | | | 146.6 | 3.782 | 9.666 | | | | |
| 4 | quadratic | 147766.8 | 0.85 | 0.47 | 0.63 | 0.74 | | | | 122.5 | 2.233 | 2.694 | 7.571 | | | |
| 5 | quadratic | 147386.6 | 0.86 | 0.59 | 0.46 | NA | 0.73 | | | 211.6 | 7.864 | 1.741 | NA | 13.43 | | |
| 6 | quadratic | 147470.7 | 0.82 | 0.6 | 0.33 | NA | NA | 0.62 | | 94.24 | 8.135 | 1.594 | NA | NA | 10.21 | |
| 7 | quadratic | 147504.9 | 0.87 | 0.66 | 0.41 | NA | NA | 0.31 | 0.52 | 226.9 | 15.5 | 4.469 | NA | NA | 1.706 | 7.64 |
| 2 | cubic | 148938.7 | 0.86 | 0.9 | | | | | | 17.32 | 3.274 | | | | | |
| 3 | cubic | 147736.3 | 0.86 | 0.88 | 0.81 | | | | | 141.5 | 3.782 | 9.647 | | | | |
| 4 | cubic | 147347 | 0.87 | 0.64 | 0.88 | 0.78 | | | | 261.9 | 9.901 | 3.852 | 16.83 | | | |
| 5 | cubic | 147425.6 | 0.84 | 0.59 | 0.48 | NA | 0.69 | | | 142.7 | 7.985 | 1.762 | NA | 11.75 | | |
| 6 | cubic | 147438.5 | 0.81 | 0.5 | 0.53 | 0.45 | NA | 0.45 | | 143.9 | 10.59 | 6.643 | 1.77 | NA | 6.299 | |
| 7 | cubic | 147383.4 | 0.88 | 0.67 | 0.44 | NA | NA | 0.38 | 0.67 | 281.4 | 18.13 | 4.796 | NA | NA | 1.735 | 14.72 |
| **Problem solving** | | | | | | | | | | | | | | | | |
| 2 | linear | 144018 | 0.89 | 0.94 | | | | | | 27.98 | 4.408 | | | | | |
| 3 | linear | 142704.7 | 0.88 | 0.92 | 0.83 | | | | | 174.7 | 4.4 | 15.19 | | | | |
| 4 | linear | 142742.9 | 0.88 | NA | 0.51 | 0.77 | | | | 170.8 | NA | 1.63 | 10.31 | | | |
| 5 | linear | 142355.6 | 0.9 | 0.51 | NA | 0.59 | 0.76 | | | 385.9 | 1.67 | NA | 9.634 | 19.01 | | |
| 6 | linear | 142526.2 | 0.89 | NA | 0.71 | 0.34 | 0.3 | 0.58 | | 322.8 | NA | 20.46 | 1.564 | 1.278 | 7.852 | |
| 7 | linear | 142361.2 | 0.89 | 0.7 | 0.44 | NA | NA | 0.35 | 0.71 | 351.8 | 31.48 | 5.636 | NA | NA | 1.571 | 19.59 |
| 2 | quadratic | 144029.2 | 0.89 | 0.94 | | | | | | 27.92 | 4.41 | | | | | |
| 3 | quadratic | 142711.7 | 0.88 | 0.92 | 0.83 | | | | | 175.5 | 4.416 | 15.09 | | | | |
| 4 | quadratic | 142750 | 0.88 | 0.44 | 0.49 | 0.76 | | | | 175.8 | 1.436 | 1.743 | 9.759 | | | |
| 5 | quadratic | 142369.9 | 0.89 | 0.6 | NA | 0.61 | 0.77 | | | 320.8 | 9.576 | NA | 1.838 | 19.25 | | |
| 6 | quadratic | 142540.7 | 0.89 | 0.72 | NA | NA | 0.32 | 0.59 | | 313.5 | 21.25 | NA | NA | 1.472 | 7.808 | |
| 7 | quadratic | 142522.8 | 0.87 | 0.67 | 0.39 | NA | 0.33 | NA | 0.51 | 247.5 | 18.83 | 4.339 | NA | 1.659 | NA | 7.806 |
| 2 | cubic | 144290.7 | 0.89 | 0.94 | | | | | | 27.4 | 4.25 | | | | | |

© 2025 Chen G et al. *JAMA Network Open*.

| n | Fixed effect | BIC | APPA for every class | | | | | | | OCC for every class | | | | | | |
|---|---|---|---|---|---|---|---|---|---|---|---|---|---|---|---|---|
| | | | 1 | 2 | 3 | 4 | 5 | 6 | 7 | 1 | 2 | 3 | 4 | 5 | 6 | 7 |
| **Problem solving** | | | | | | | | | | | | | | | | |
| 3 | cubic | 142720.7 | 0.88 | 0.92 | 0.83 | | | | | 176.2 | 4.418 | 15.06 | | | | |
| 4 | cubic | 142360.2 | 0.89 | 0.62 | 0.9 | 0.79 | | | | 307.5 | 9.634 | 4.297 | 20.88 | | | |
| 5 | cubic | 142388.6 | 0.89 | NA | 0.5 | 0.58 | 0.76 | | | 280.4 | NA | 1.697 | 8.617 | 18.29 | | |
| 6 | cubic | 142678 | 0.86 | 0.67 | 0.29 | NA | 0.39 | 0.54 | | 197 | 14.91 | 1.626 | NA | 1.6 | 7.246 | |
| 7 | cubic | 142482.3 | 0.87 | 0.67 | 0.42 | NA | NA | 0.33 | 0.49 | 266 | 21.6 | 5.574 | NA | NA | 1.588 | 7.537 |
| **Personal/social** | | | | | | | | | | | | | | | | |
| 2 | linear | 148839.4 | 0.91 | 0.98 | | | | | | 152.9 | 3.967 | | | | | |
| 3 | linear | 146730.8 | 0.86 | 0.89 | 0.76 | | | | | 58.4 | 4.213 | 10.09 | | | | |
| 4 | linear | 146332.8 | 0.9 | 0.68 | 0.88 | 0.79 | | | | 528.2 | 8.347 | 4.237 | 22.7 | | | |
| 5 | linear | 146371.7 | 0.91 | 0.64 | NA | 0.53 | 0.77 | | | 526.9 | 6.874 | NA | 1.908 | 20.24 | | |
| 6 | linear | 146363.3 | 0.9 | 0.44 | 0.6 | 0.51 | NA | 0.74 | | 449.7 | 3.86 | 13.32 | 1.973 | NA | 19.61 | |
| 7 | linear | 146394.9 | 0.89 | 0.55 | 0.3 | NA | NA | 0.55 | 0.7 | 466.6 | 11.17 | 3.698 | NA | NA | 1.935 | 22.23 |
| 2 | quadratic | 148848.8 | 0.91 | 0.98 | | | | | | 152.9 | 3.964 | | | | | |
| 3 | quadratic | 146740 | 0.86 | 0.89 | 0.76 | | | | | 58.56 | 4.214 | 10.09 | | | | |
| 4 | quadratic | 146343 | 0.9 | 0.88 | 0.68 | 0.79 | | | | 493.4 | 4.238 | 8.339 | 21.79 | | | |
| 5 | quadratic | 146394.7 | 0.87 | 0.61 | 0.48 | NA | 0.74 | | | 287.1 | 6.405 | 1.846 | NA | 16.89 | | |
| 6 | quadratic | 146409.5 | 0.88 | 0.59 | NA | 0.35 | 0.46 | 0.72 | | 309.4 | 8.879 | NA | 3.037 | 1.937 | 17.26 | |
| 7 | quadratic | 146404.1 | 0.89 | 0.73 | NA | NA | 0.52 | 0.41 | 0.64 | 508.8 | 26.47 | NA | NA | 4.351 | 1.842 | 16.6 |
| 2 | cubic | 148478.9 | 0.86 | 0.92 | | | | | | 18.6 | 3.576 | | | | | |
| 3 | cubic | 146765.7 | 0.86 | 0.89 | 0.78 | | | | | 70.44 | 4.19 | 9.862 | | | | |
| 4 | cubic | 146363.7 | 0.88 | 0.88 | 0.67 | 0.78 | | | | 326.5 | 4.237 | 8.129 | 19.9 | | | |
| 5 | cubic | 146405.9 | 0.88 | 0.63 | 0.57 | NA | 0.75 | | | 290.6 | 6.913 | 1.987 | NA | 17.62 | | |
| 6 | cubic | 146444.7 | 0.87 | 0.59 | 0.32 | 0.41 | NA | 0.71 | | 250.8 | 7.63 | 2.526 | 1.942 | NA | 15.66 | |
| 7 | cubic | 146427.7 | 0.89 | 0.57 | 0.27 | 0.32 | 0.59 | NA | 0.71 | 450 | 10.63 | 3.178 | 4.138 | 2.006 | NA | 25.47 |

[a] Linear, quadratic, or cubic effects of age, at which the ASQ-3 were completed, were applied to the fixed effect model
APPA, average maximum posterior probability of assignments; OCC, odds of correct classification; BIC, Bayesian information criteria; NA, not available.

© 2025 Chen G et al. *JAMA Network Open*.

**eTable 6. Congenital abnormalities excluded in the sensitivity analysis**

| Type | Congenital abnormality |
|---|---|
| Cranial abnormalities | anencephaly, microcephaly, hydrocephalus, craniotabes, holoprosencephaly, agenesis of corpus callosum |
| Ocular abnormalities | ablepharon, microphthalmia, anophthalmia, cataract |
| Optic abnormalities | hearing impairment, microtia, atresia of auditory canal, cryptotia, low-set ear |
| Oral abnormalities | cleft lip, cleft palate, cleft lip and palate, prosoposchisis, congenital tooth |
| Chromosomal abnormalities | Down syndrome, Trisomy 18, Trisomy 13, Klinefelter syndrome, Turner syndrome, Prader-Willi syndrome, Trisomy 1q |
| Limb abnormalities | polydactyly, zygodactyly, split-hand malformation, split-foot malformation |
| Thoracic abnormalities | congenital diaphragmatic hernia, pulmonary sequestration, congenital cystic adenomatoid malformation, pulmonary hypoplasia, congenital heart disease, arrhythmia |
| Abdominal abnormalities | umbilical hernia, gastroschisis, congenital esophageal atresia, duodenal atresia, atresia of small intestine, atresia of anus/ atresia ani, anorectal anomaly, inguinal hernia |
| Genitourinary abnormalities | congenital hydronephrosis, multicystic dysplastic kidney, renal agenesis, hypospadias, cryptorchid, nonpalpable testis, bladder exstrophy, cloacal exstrophy, clitoromegaly, vaginal atresia, atypical genitalia |
| Epidermal abnormalities | ≥ 6 large melasma (brown, black, red, white), angioma/hemangioma, epidermolysis bullosa, incontinentia pigmenti |
| Musculo-skeletal abnormalities | myelomeningocele, spina bifida, chondrodysplasia, achondroplasia, osteogenesis imperfecta, arthrogryposis multiplex congenita, hypotonia |
| Others | conjoined fetus, amniotic band syndrome |

© 2025 Chen G et al. *JAMA Network Open*.

**eTable 7. Number of ASQ-3 responses in the analysis population (n=14023)**

| Time point (Unit: month) | ASQ-3 domain | | | | |
|---|---|---|---|---|---|
| | Communication | Gross motor | Fine motor | Problem solving | Personal/social |
| 6 | 13940 | 13942 | 13907 | 13899 | 13912 |
| 12 | 12470 | 12469 | 12454 | 12402 | 12427 |
| 24 | 11396 | 11397 | 11397 | 11349 | 11372 |
| 42 | 10249 | 10262 | 10227 | 10199 | 10251 |
| 48 | 9324 | 9320 | 9321 | 9311 | 9333 |

ASQ-3, Ages and Stages Questionnaires, third edition

© 2025 Chen G et al. *JAMA Network Open*.

## eTable 8-1. Relative risks in child developmental patterns across HDP and subtypes in complete cases

| Domain | HDP | | GH | | PE | | PEEO | | PELO | |
|---|---|---|---|---|---|---|---|---|---|---|
| | Adjusted RR (95% CI)[a] | P-value[b] | Adjusted RR (95% CI)[a] | P-value[b] | Adjusted RR (95% CI)[a] | P-value[b] | Adjusted RR (95% CI)[a] | P-value[b] | Adjusted RR (95% CI)[a] | P-value[b] |
| **Communication** | | | | | | | | | | |
| Catch-up vs normal | 1.06 (0.94-1.19) | 0.998 | 1.06 (0.88-1.28) | 0.990 | 1.13 (0.90-1.41) | 0.766 | 1.52 (1.00-2.32) | 0.562 | 1.02 (0.79-1.34) | 0.860 |
| Delay vs normal | 1.11 (0.91-1.35) | 0.998 | 1.00 (0.73-1.37) | 0.990 | 1.38 (0.99-1.94) | 0.739 | 1.84 (0.98-3.44) | 0.562 | 1.26 (0.85-1.87) | 0.860 |
| Catch-up vs delay | 0.98 (0.87-1.11) | 0.998 | 1.02 (0.85-1.23) | 0.990 | 0.93 (0.74-1.17) | 0.766 | 0.95 (0.63-1.46) | 0.959 | 0.93 (0.71-1.21) | 0.860 |
| **Gross motor** | | | | | | | | | | |
| Catch-up vs normal | 1.06 (0.92-1.22) | 0.998 | 0.98 (0.79-1.23) | 0.990 | 1.15 (0.90-1.49) | 0.766 | 1.42 (0.87-2.32) | 0.959 | 1.08 (0.81-1.45) | 0.860 |
| Delay vs normal | 1.24 (1.04-1.49) | 0.289 | 1.25 (0.95-1.65) | 0.990 | 1.46 (1.06-2.02) | 0.303 | 1.74 (0.93-3.25) | 0.745 | 1.39 (0.96-2.02) | 0.860 |
| Catch-up vs delay | 0.94 (0.82-1.08) | 0.998 | 0.91 (0.72-1.13) | 0.990 | 0.92 (0.71-1.19) | 0.766 | 0.91 (0.55-1.49) | 0.959 | 0.92 (0.69-1.24) | 0.860 |
| **Fine motor** | | | | | | | | | | |
| Catch-up vs normal | 1.08 (0.96-1.21) | 0.998 | 1.16 (0.98-1.37) | 0.990 | 1.10 (0.89-1.36) | 0.766 | 1.17 (0.75-1.82) | 0.959 | 1.08 (0.85-1.38) | 0.860 |
| Delay vs normal | 1.06 (0.76-1.47) | 0.998 | 0.77 (0.42-1.41) | 0.990 | 1.64 (0.97-2.76) | 0.766 | 2.39 (0.98-5.80) | 0.562 | 1.41 (0.75-2.66) | 0.860 |
| Catch-up vs delay | 1.01 (0.90-1.13) | 0.998 | 1.04 (0.88-1.23) | 0.990 | 0.96 (0.77-1.19) | 0.766 | 0.90 (0.58-1.39) | 0.959 | 0.98 (0.77-1.25) | 0.860 |
| **Problem solving** | | | | | | | | | | |
| Catch-up vs normal | 1.09 (0.97-1.24) | 0.998 | 1.10 (0.91-1.33) | 0.990 | 1.18 (0.94-1.49) | 0.766 | 1.65 (1.09-2.48) | 0.268 | 1.06 (0.80-1.39) | 0.860 |
| Delay vs normal | 1.00 (0.73-1.38) | 0.998 | 0.97 (0.58-1.61) | 0.990 | 1.75 (1.08-2.83) | 0.313 | 2.40 (0.99-5.83) | 0.562 | 1.59 (0.91-2.77) | 0.860 |
| Catch-up vs delay | 1.01 (0.89-1.14) | 0.998 | 1.01 (0.83-1.22) | 0.990 | 0.93 (0.74-1.18) | 0.766 | 0.95 (0.63-1.43) | 0.959 | 0.93 (0.71-1.22) | 0.860 |
| **Personal/social** | | | | | | | | | | |
| Catch-up vs normal | 1.06 (0.94-1.20) | 0.998 | 1.09 (0.90-1.32) | 0.990 | 1.15 (0.92-1.45) | 0.766 | 1.54 (1.01-2.35) | 0.562 | 1.05 (0.80-1.37) | 0.860 |
| Delay vs normal | 1.06 (0.85-1.32) | 0.998 | 0.98 (0.69-1.39) | 0.990 | 1.31 (0.89-1.93) | 0.766 | 1.48 (0.66-3.32) | 0.959 | 1.27 (0.82-1.96) | 0.860 |
| Catch-up vs delay | 0.99 (0.87-1.12) | 0.998 | 1.01 (0.83-1.22) | 0.990 | 0.97 (0.77-1.22) | 0.766 | 1.01 (0.66-1.54) | 0.959 | 0.95 (0.72-1.25) | 0.860 |

[a]Reference group: HDP-unaffected. Adjusted for maternal age at pregnancy (linear and quadratic effects), family annual income, maternal educational attainment, maternal pre-pregnancy body mass index, parity, gestational diabetes mellitus prevalence, maternal tobacco use during pregnancy, maternal alcohol use during pregnancy, maternal folic acid intake during pregnancy, and the child’s sex.
[b]adjusted for multiple comparisons using the Hochberg procedure.
HDP, hypertensive disorders of pregnancy; PE, preeclampsia; GH, gestational hypertension; EO, early onset; LO, late onset; RR, risk ratio; CI, confidence interval.

© 2025 Chen G et al. *JAMA Network Open*.

**eTable 8-2. Relative risks in child developmental patterns across HDP subtypes after excluding congenital abnormalities**

| Domain | HDP | | GH | | PE | | PEEO | | PELO | |
|---|---|---|---|---|---|---|---|---|---|---|
| | Adjusted RR (95% CI)[a] | P-value[b] | Adjusted RR (95% CI)[a] | P-value[b] | Adjusted RR (95% CI)[a] | P-value[b] | Adjusted RR (95% CI)[a] | P-value[b] | Adjusted RR (95% CI)[a] | P-value[b] |
| **Communication** | | | | | | | | | | |
| Catch-up vs normal | 1.05 (0.93-1.17) | 0.999 | 1.01 (0.85-1.21) | 0.926 | 1.15 (0.93-1.42) | 0.724 | 1.56 (1.07-2.28) | 0.186 | 1.03 (0.80-1.33) | 0.953 |
| Delay vs normal | 1.16 (0.97-1.39) | 0.999 | 1.02 (0.76-1.37) | 0.926 | 1.42 (1.03-1.95) | 0.387 | 2.02 (1.16-3.51) | 0.155 | 1.23 (0.84-1.81) | 0.953 |
| Catch-up vs delay | 0.96 (0.85-1.08) | 0.999 | 0.99 (0.83-1.18) | 0.926 | 0.93 (0.75-1.15) | 0.724 | 0.93 (0.63-1.36) | 0.855 | 0.93 (0.72-1.20) | 0.953 |
| **Gross motor** | | | | | | | | | | |
| Catch-up vs normal | 1.05 (0.92-1.20) | 0.999 | 0.97 (0.79-1.20) | 0.926 | 1.20 (0.95-1.53) | 0.724 | 1.64 (1.08-2.51) | 0.186 | 1.08 (0.81-1.43) | 0.953 |
| Delay vs normal | 1.25 (1.05-1.48) | 0.194 | 1.19 (0.91-1.56) | 0.926 | 1.57 (1.17-2.13) | 0.046 | 2.01 (1.16-3.48) | 0.155 | 1.45 (1.02-2.07) | 0.580 |
| Catch-up vs delay | 0.94 (0.82-1.07) | 0.999 | 0.92 (0.75-1.14) | 0.926 | 0.91 (0.72-1.16) | 0.724 | 0.93 (0.61-1.42) | 0.855 | 0.91 (0.68-1.20) | 0.953 |
| **Fine motor** | | | | | | | | | | |
| Catch-up vs normal | 1.10 (0.99-1.22) | 0.999 | 1.14 (0.97-1.33) | 0.926 | 1.11 (0.91-1.36) | 0.724 | 1.24 (0.84-1.83) | 0.855 | 1.07 (0.85-1.35) | 0.953 |
| Delay vs normal | 1.16 (0.85-1.59) | 0.999 | 0.80 (0.45-1.43) | 0.926 | 1.76 (1.06-2.92) | 0.381 | 2.71 (1.20-6.12) | 0.184 | 1.45 (0.77-2.74) | 0.953 |
| Catch-up vs delay | 1.00 (0.90-1.11) | 0.999 | 1.03 (0.88-1.21) | 0.926 | 0.96 (0.78-1.17) | 0.724 | 0.91 (0.61-1.34) | 0.855 | 0.98 (0.77-1.23) | 0.953 |
| **Problem solving** | | | | | | | | | | |
| Catch-up vs normal | 1.10 (0.98-1.24) | 0.999 | 1.04 (0.87-1.25) | 0.926 | 1.16 (0.93-1.44) | 0.724 | 1.64 (1.13-2.38) | 0.138 | 1.01 (0.77-1.32) | 0.953 |
| Delay vs normal | 1.07 (0.79-1.45) | 0.999 | 0.89 (0.54-1.47) | 0.926 | 1.84 (1.16-2.91) | 0.126 | 2.88 (1.35-6.13) | 0.093 | 1.55 (0.88-2.70) | 0.953 |
| Catch-up vs delay | 1.01 (0.90-1.13) | 0.999 | 1.02 (0.85-1.22) | 0.926 | 0.93 (0.74-1.16) | 0.724 | 0.92 (0.63-1.34) | 0.855 | 0.93 (0.71-1.22) | 0.953 |
| **Personal/social** | | | | | | | | | | |
| Catch-up vs normal | 1.06 (0.94-1.19) | 0.999 | 1.05 (0.88-1.26) | 0.926 | 1.15 (0.93-1.44) | 0.724 | 1.55 (1.06-2.27) | 0.186 | 1.03 (0.79-1.34) | 0.953 |
| Delay vs normal | 1.12 (0.91-1.38) | 0.999 | 1.02 (0.73-1.41) | 0.926 | 1.38 (0.96-2.00) | 0.724 | 1.49 (0.70-3.14) | 0.855 | 1.35 (0.89-2.05) | 0.953 |
| Catch-up vs delay | 0.98 (0.87-1.11) | 0.999 | 0.99 (0.83-1.19) | 0.926 | 0.96 (0.77-1.20) | 0.724 | 1.04 (0.71-1.52) | 0.855 | 0.93 (0.71-1.21) | 0.953 |

[a] Reference group: HDP-unaffected. Adjusted for maternal age at pregnancy (linear and quadratic effects), family annual income, maternal educational attainment, maternal pre-pregnancy body mass index, parity, gestational diabetes mellitus prevalence, maternal tobacco use during pregnancy, maternal alcohol use during pregnancy, maternal folic acid intake during pregnancy, and the child's sex.
[b] adjusted for multiple comparisons using the Hochberg procedure.
HDP, hypertensive disorders of pregnancy; PE, preeclampsia; GH, gestational hypertension; EO, early onset; LO, late onset; RR, risk ratio; CI, confidence interval.

© 2025 Chen G et al. *JAMA Network Open*.

## eTable 8-3. Differences in longitudinal trends in child development by HDP subtypes, estimated by linear mix model

| Domain | HDP | | GH | | PE | | PEEO | | PELO | |
|---|---|---|---|---|---|---|---|---|---|---|
| | Estimate (95% CI)[a] | P-value[b] | Estimate (95% CI)[a] | P-value[b] | Estimate (95% CI)[a] | P-value[b] | Estimate (95% CI)[a] | P-value[b] | Estimate (95% CI)[a] | P-value[b] |
| **Communication** | | 0.227 | | 0.871 | | 0.040 | | 0.003 | | 0.727 |
| exposure | -0.01 (-0.06-0.04) | | -0.01 (-0.08-0.07) | | -0.06 (-0.16-0.04) | | -0.24 (-0.44--0.05) | | 0.00 (-0.11-0.11) | |
| exposure:timepoint | 0.00 (0.00-0.00) | | 0.00 (0.00-0.00) | | 0.00 (-0.01-0.00) | | 0.00 (-0.01-0.01) | | 0.00 (-0.01-0.00) | |
| **Gross motor** | | 0.227 | | 0.871 | | 0.096 | | 0.002 | | 0.727 |
| exposure | -0.05 (-0.10-0.01) | | -0.06 (-0.15-0.02) | | -0.09 (-0.19-0.02) | | -0.40 (-0.62--0.19) | | 0.01 (-0.11-0.13) | |
| exposure:timepoint | 0.00 (0.00-0.00) | | 0.00 (0.00-0.00) | | 0.00 (0.00-0.00) | | 0.00 (0.00-0.01) | | 0.00 (-0.01-0.00) | |
| **Fine motor** | | 0.227 | | 0.871 | | 0.074 | | 0.002 | | 0.727 |
| exposure | -0.01 (-0.06-0.05) | | -0.02 (-0.10-0.06) | | -0.08 (-0.19-0.02) | | -0.40 (-0.61--0.18) | | 0.01 (-0.10-0.13) | |
| exposure:timepoint | 0.00 (0.00-0.00) | | 0.00 (0.00-0.00) | | 0.00 (0.00-0.00) | | 0.01 (0.00-0.01) | | 0.00 (-0.01-0.00) | |
| **Problem solving** | | 0.357 | | 0.871 | | 0.044 | | <0.001 | | 0.727 |
| exposure | -0.04 (-0.10-0.01) | | -0.03 (-0.11-0.06) | | -0.15 (-0.26--0.05) | | -0.52 (-0.72--0.31) | | -0.04 (-0.16-0.07) | |
| exposure:timepoint | 0.00 (0.00-0.00) | | 0.00 (0.00-0.00) | | 0.00 (0.00-0.01) | | 0.01 (0.00-0.02) | | 0.00 (0.00-0.00) | |
| **Personal/social** | | 0.161 | | 0.871 | | 0.013 | | <0.001 | | 0.727 |
| exposure | -0.06 (-0.12--0.01) | | -0.05 (-0.13-0.03) | | -0.18 (-0.28--0.08) | | -0.45 (-0.65--0.24) | | -0.10 (-0.21-0.01) | |
| exposure:timepoint | 0.00 (0.00-0.00) | | 0.00 (0.00-0.00) | | 0.00 (0.00-0.01) | | 0.01 (0.00-0.01) | | 0.00 (0.00-0.01) | |

[a]Adjusted for maternal age at pregnancy (linear and quadratic effects), family annual income, maternal educational attainment, maternal pre-pregnancy body mass index, parity, gestational diabetes mellitus prevalence, maternal tobacco use during pregnancy, maternal alcohol use during pregnancy, maternal folic acid intake during pregnancy, and the child's sex.
[b]Omnibus p values from likelihood ratio tests (D2 method, combined across imputations) were adjusted for multiple comparisons using the Hochberg procedure.
HDP, hypertensive disorders of pregnancy; PE, preeclampsia; GH, gestational hypertension; EO, early onset; LO, late onset; CI, confidence interval.

© 2025 Chen G et al. *JAMA Network Open*.

## eTable 9. Baseline characteristics among participants included and excluded by ASQ-3 response

| Characteristics | Mother-child pairs, No. (%) | | *P*-value |
|---|---|---|---|
| | Excluded (n=7866) | Included (n=14044) | |
| **Maternal age, year, mean (SD)** | 31.5 (5.2) | 32.5 (4.8) | <0.001 |
| **Family income, JPY/year** | | | <0.001 |
| < 4 000 000 | 2713 (40.9) | 4614 (34.7) | |
| ≥ 4 000 000, < 6 000 000 | 2051 (30.9) | 4415 (33.2) | |
| ≥ 6 000 000 | 1865 (28.1) | 4257 (32.0) | |
| **Maternal educational level** | | | <0.001 |
| High school or lower | 445 (38.3) | 4099 (32.4) | |
| Junior or vocational college | 437 (37.6) | 4911 (38.8) | |
| University or higher | 281 (24.2) | 3645 (28.8) | |
| **Maternal pre-pregnancy BMI[a], kg/m$^2$** | | | <0.001 |
| < 18.5 | 5564 (71.7) | 10293 (74.5) | |
| ≥ 18.5, < 25 | 1065 (13.7) | 1821 (13.2) | |
| ≥ 25 | 1134 (14.6) | 1708 (12.4) | |
| **Parity** | | | 0.008 |
| Multipara | 4292 (54.6) | 7397 (52.8) | |
| **Gestational diabetes mellitus prevalence** | | | 0.093 |
| Yes | 214 (2.7) | 329 (2.3) | |
| **Maternal tobacco use** | | | <0.001 |
| Yes | 1692 (23.8) | 1880 (13.5) | |
| **Maternal alcohol use** | | | 0.049 |
| Yes | 1461 (20.6) | 3033 (21.8) | |
| **Maternal folic acid intake** | | | <0.001 |
| No during pregnancy | 3211 (44.9) | 5246 (37.7) | |
| Yes during pregnancy | 3010 (42.1) | 6165 (44.3) | |
| Yes before and during pregnancy | 936 (13.1) | 2494 (17.9) | |
| **Preterm Birth** | | | 0.008 |
| Yes | 469 (6.0) | 717 (5.1) | |
| **Child sex** | | | 0.461 |
| Female | 3829 (48.7) | 6762 (48.1) | |

[a]BMI was calculated by dividing the pre-pregnancy weight (kg) by the square of height (m$^2$). JPY, Japanese Yen; BMI, body mass index

© 2025 Chen G et al. *JAMA Network Open*.
