# Supplementary material for: Child Developmental Patterns by Age 4 Years Across Subtypes of Hypertensive Disorders of Pregnancy
Source: JAMA Netw Open. 2025 Nov 26;8(11):e2545719. doi: 10.1001/jamanetworkopen.2025.45719 (PMC12658654; doi:10.1001/jamanetworkopen.2025.45719)
Supplement: Supplement 2. — Data Sharing Statement [file jamanetwopen-e2545719-s002.pdf]

Data Sharing Statement

Chen. Child Developmental Patterns by Age 4 Years Across Subtypes of Hypertensive Disorders of Pregnancy. *JAMA Netw Open*. Published November 26, 2025.
doi:10.1001/jamanetworkopen.2025.45719

Data
Data available: No

Additional Information
Explanation for why data not available: The datasets generated and/or analyzed in the current study are available from the corresponding author upon reasonable request.
